# Bioinformatics Analysis of Bacterial Annexins – Putative Ancestral Relatives of Eukaryotic Annexins

**DOI:** 10.1371/journal.pone.0085428

**Published:** 2014-01-16

**Authors:** Praveen Kumar Kodavali, Małgorzata Dudkiewicz, Sławomir Pikuła, Krzysztof Pawłowski

**Affiliations:** 1 Department of Biochemistry, Nencki Institute of Experimental Biology, Polish Academy of Sciences, Warsaw, Poland; 2 Faculty of Agriculture and Biology, Warsaw University of Life Sciences, Warsaw, Poland; UC Irvine, United States of America

## Abstract

Annexins are Ca^2+^-binding, membrane-interacting proteins, widespread among eukaryotes, consisting usually of four structurally similar repeated domains. It is accepted that vertebrate annexins derive from a double genome duplication event. It has been postulated that a single domain annexin, if found, might represent a molecule related to the hypothetical ancestral annexin. The recent discovery of a single-domain annexin in a bacterium, *Cytophaga hutchinsonii*, apparently confirmed this hypothesis. Here, we present a more complex picture. Using remote sequence similarity detection tools, a survey of bacterial genomes was performed in search of annexin-like proteins. In total, we identified about thirty annexin homologues, including single-domain and multi-domain annexins, in seventeen bacterial species. The thorough search yielded, besides the known annexin homologue from *C. hutchinsonii*, homologues from the *Bacteroidetes/Chlorobi* phylum, from *Gemmatimonadetes*, from beta- and delta-*Proteobacteria*, and from *Actinobacteria*. The sequences of bacterial annexins exhibited remote but statistically significant similarity to sequence profiles built of the eukaryotic ones. Some bacterial annexins are equipped with additional, different domains, for example those characteristic for toxins. The variation in bacterial annexin sequences, much wider than that observed in eukaryotes, and different domain architectures suggest that annexins found in bacteria may actually descend from an ancestral bacterial annexin, from which eukaryotic annexins also originate. The hypothesis of an ancient origin of bacterial annexins has to be reconciled with the fact that remarkably few bacterial strains possess annexin genes compared to the thousands of known bacterial genomes and with the patchy, anomalous phylogenetic distribution of bacterial annexins. Thus, a massive annexin gene loss in several bacterial lineages or very divergent evolution would appear a likely explanation. Alternative evolutionary scenarios, involving horizontal gene transfer between bacteria and protozoan eukaryotes, in either direction, appear much less likely. Altogether, current evidence does not allow unequivocal judgement as to the origin of bacterial annexins.

## Introduction

As of January 19th, 2013, 12795 PubMed articles mention annexins in the title or abstract, making them one of the well studied protein families. Annexins are a multi-functional protein family, widespread in eukaryotes. The archetypical annexin proteins (e.g. the vertebrate ones) are made of four repeated domains (so-called annexin repeats) of approximately 70 amino acid residues [1]. Although the four domains usually share 40–50% sequence identity, some studies reveal that domain III is more divergent than the other domains, suggesting that the other domains might have arisen from a monomeric domain III by gene duplications [2]–[4]. The main molecular properties of annexins comprise calcium ion binding and calcium-dependent and independent membrane binding [5], [6]. The diverse biological functions of annexins include regulation of membrane trafficking and calcium homeostasis, actin and integrin binding, ATPase, GTPase, and peroxidase activity [7]–[10]. Gene duplications at different periods during eukaryotic evolution have contributed to the diversity in the annexin sequence, structure and function [11], [12].

Molecular phylogenetic analysis suggested that plant and protist annexins evolved prior to their animal counterparts from a common ancestor [13]. Evidence of the ancient presence of annexins in eukaryotes came with the discovery of annexin homologues, giardins, in the protozoan *Giardia lamblia* [4], [13]. A single domain annexin from the bacterium *Cytophaga hutchinsonii* has been reported by Fernandez, Morgan and co-workers to be the most evolutionarily distant annexin discovered so far, however its phylogenetic relationship with other annexins is yet to be determined [14]. The ancient origin of annexins has been further documented by a recent survey of eukaryotic annexins showing their presence in four out of six major eukaryotic clades, including the *Stramenopiles-Alveolata-Rhizaria* clade [15]. In the current study, we identified annexins in multiple bacteria and performed comprehensive bioinformatics analyses to probe their possible evolutionary origin and relationship to their eukaryotic counterparts.

## Results and Discussion

### A number of bacterial strains possess annexin homologues

Although annexins have been described as eukaryote-specific proteins, a single domain annexin protein has been reported in a bacterium of the *Bacteroidetes/Chlorobi* phylum, *Cytophaga hutchinsonii* [14]. This has prompted us to explore protein sequence databases in search of more bacterial annexin homologues. The search was performed using: a) iterative sequence searches using the HMM-based Jackhmmer and HHsenser tools, b) inspection of annexin annotations for bacterial proteins contained in the NCBI Protein database and in the Pfam protein domain database, c) iterative PSI-Blast searches starting from selected eukaryotic annexin domain sequences and novel bacterial ones. Any “annexin-like” annotations and weak sequence similarity hits were checked using sensitive protein sequence comparison tools, FFAS and HHpred [16], [17].

A survey of bacterial annexins, performed by the Jackhmmer tool using the definition of the annexin domain from the Pfam database (see Methods), brought twelve potential annexin proteins from eight bacterial species (See Table S1). These domains exhibited statistically significant sequence similarity to known eukaryotic annexins, as judged by the E-values reported by HHpred and hmmscan algorithms (see Table S1). Additionally, using a more “greedy” HHsenser iterative search, sixteen bacterial annexin proteins were identified after confirming the protein sequence similarity to annexins by hmmscan (HMMER package [18]), and discarding likely false positives (See Table S1). Yet additional bacterial annexins were identified by BLAST searches in the NCBI nr database using other bacterial annexin domains as queries. In total, thirty four putative bacterial annexin proteins containing forty seven annexin domains were identified in seventeen different bacterial species (Table S1). Although the sequence similarities observed between bacterial annexin domains and their closest eukaryotic counterparts were significant as judged by statistical criteria, they were not very high in terms of sequence identity, typically amounting to about 40% in alignments about 50 residues long.

**Figure 1:**
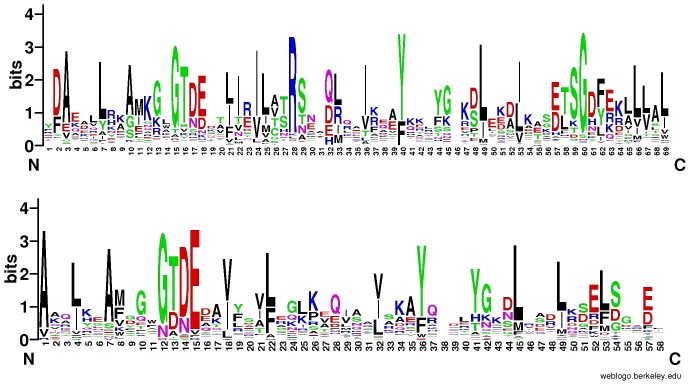
Sequence logos (weblogo.berkeley.edu) showing amino-acid residue conservation in eukaryotic annexins (upper logo), and bacterial annexins (lower logo). For the bacterial annexins, alignment from Fig. 3 used.

Although as many as 48 bacterial proteins in the curated RefSeq database at NCBI [19] are annotated as “annexin”, “annexin-like” or “putative annexin”, only 14 of them matched our list, while the remaining 34 could not be confirmed by sequence comparison methods and most likely represent annotation artefacts. It has to be pointed out that here we used rigorous Hidden Markov Model sequence comparison algorithms and applied strict decision criteria to assign annexin domain similarity to bacterial proteins. The discovered bacterial annexin domains possess sequence features similar to the eukaryotic ones (see Fig. 1).

Using the same approach, no annexins were found in *Archaea* or viruses.

### Protein domain architectures and genomic neighbourhoods of bacterial annexins

**Figure 2:**
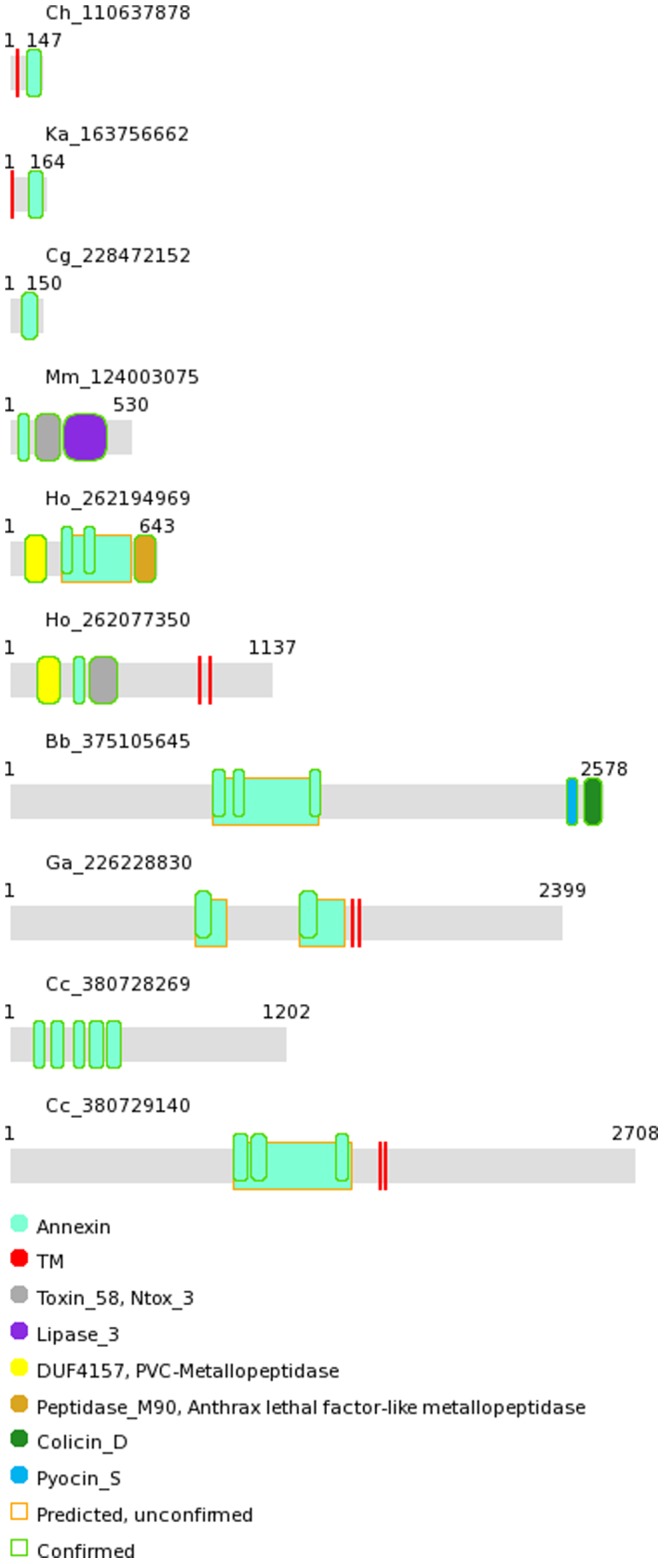
Domain composition of bacterial annexins. Domain architectures for bacterial annexins. HMMER3 and HHpred assignments of Pfam domains shown, as well as transmembrane region (TM) predictions. Broader rectangles with gold edges indicate weak similarities to full annexin tetrads (see also Fig. S3). Proteins identified by NCBI gi identifiers, preceded by species acronyms (see Fig. 3 caption).

Bacterial proteins in which annexin domains were identified differed markedly in length (See Table S1). Alongside annexin domains, other domains were found, namely putative zinc-dependent metalloproteases (Pfam domains DUF4157 and Peptidase_M90) in annexins from *Haliangium ochraceum*, and a lipase domain in annexin from *Microscilla marina*. Also, different toxin domains were identified in annexins from *Burkholderiales* bacterium JOSHI_001, *H. ochraceum* and *M. marina*. Careful examination of the remaining regions of bacterial annexin sequences by sequence comparison tools, HHpred and FFAS, revealed additional annexin domains in some bacterial proteins (See Fig. 2). Thus, two such domains were found in annexins from the *Gemmatimonadetes* bacterium, *Gemmatimonas aurantiaca*, from delta-proteobacterium *Haliangium ochraceum* and from actinobacterium *Rhodococcus imtechensis*. Three domains were present in annexins from beta-proteobacterium *Burkholderiales* bacterium JOSHI_001, and delta-proteobacteria *Corallococcus coralloides* DSM 2259 and *Myxococcus stipitatus* DSM 14675. Finally, five domains were found in another protein from *C. coralloides* DSM 2259. It cannot be excluded that further annexin domains that have diverged in sequence beyond easy recognition, are present in bacterial annexins. For example, in annexins from *Burkholderiales* and *Corallococcus*, there are regions between the identified annexin domains that could harbour those additional, difficult to identify annexins domains (see Fig. 2). Indeed, a HHpred analysis of some wider regions of bacterial annexin proteins showed sequence similarities to annexin tetrads (units of four repeated domains) – See Fig. 2 and Fig. S3. Albeit the similarities to full annexin tetrads did not reach high statistical significance, they were suggestive of the presence of four-domain units similar to those well-known in eukaryotes.

The presence of multi-domain annexins in some bacteria and the probable presence of such proteins in some others suggest that, just like in eukaryotic annexins, the functional and structural units may be formed by pairs or fours of domains. In cases where other numbers of annexin domains per bacterial protein are found (e.g. three or five), the ‘missing’ domains might have simply diverged beyond recognition. This is further corroborated by the genomic arrangement of annexin proteins in genomes of members of the *Bacteroidetes/Chlorobi* phylum, the flavobacteria *Kordia algicida* OT-1, *Aquimarina agarilytica* ZC1 and *Flavobacteriaceae* bacterium HQM9. In these, pairs of relatively short annexin proteins are immediate genomic neighbours. One such annexin pair is found in HQM9 and *A. agarilytica*, and three – in *K. algicida*. Such conservation of genomic adjacency has been used as a predictive factor in predicting functional relationships, protein-protein interactions in particular [20], [21].

The unique domain architectures of bacterial annexins, the presence of specific structural domains (e.g. enzymatic ones) and transmembrane regions suggest ancient origin of bacterial annexins. Of note, no such additional domains have been described in eukaryotic annexins.

### Conserved sequence features in bacterial annexins

**Figure 3:**
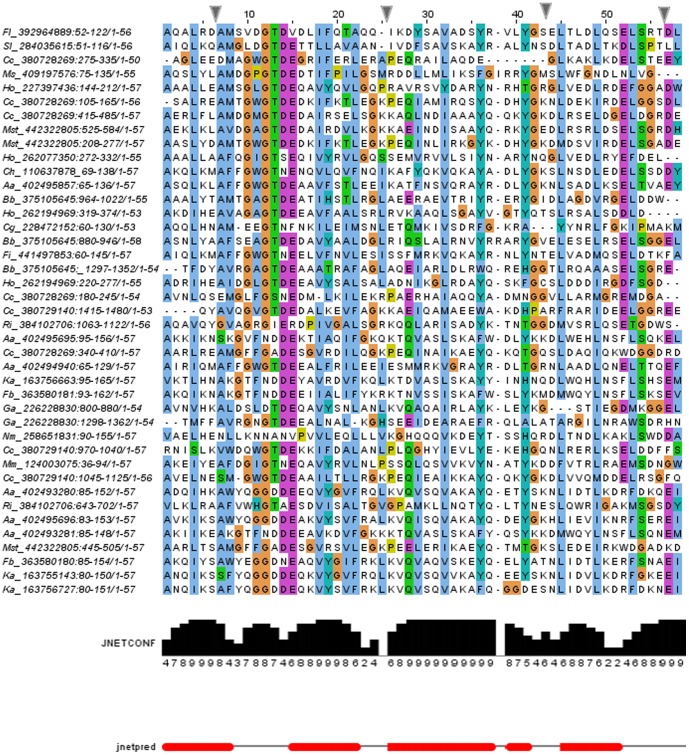
Multiple sequence alignment (Promals3D) of selected bacterial annexin domains. Alignment is manually edited in the GxGTDE region, sequence redundancy at 70% identity removed. Alignment columns containing mostly gaps hidden (as marked by blue markers above). Secondary structure prediction shown (Jnet algorithm), red bars represent alpha-helices. The JnetConf histogram represents the confidence of secondary structure prediction at each position. Proteins identified by NCBI gi identifiers preceded by species acronyms: Aa, *Aquimarina agarilytica* ZC1, Bb, *Burkholderiales* bacterium HQ_001, Cc, *Corallococcus coralloides* DSM 2259, Cg, *Capnocytophaga gingivalis* ATCC 33624, Ch, *Cytophaga hutchinsonii* ATCC 33406, Fb, *Flavobacteriaceae* bacterium HQM9, Fi, *Fulvivirga imtechensis* AK7, Ga, *Gemmatimonas aurantiaca* T-27, Ho, *Haliangium ochraceum* DSM 14365, Ko, *Kordia algicida* OT-1, Mm, *Microscilla marina* ATCC 23134, Ms, *Marinilabilia salmonicolor* JCM 21150, Mst, *Myxococcus stipitatus* DSM 14675, Nm, *Nakamurella multipartita* DSM 44233, Ri, *Rhodococcus imtechensis* RKJ300, Sl, *Spirosoma linguale* DSM 74.

Annexin domains are made up of five conserved alpha-helices, conventionally named A-E, that are packed in a conserved arrangement [22], [23]. Multiple sequence alignment of bacterial annexin domains, when used for secondary structure prediction, provides a picture consistent with annexin structure (see Fig. 3). The most characteristic annexin sequence motif is GxGTD, forming a type II Ca^2+^ -binding motif between helices A and B. This feature is very strongly conserved in bacterial annexins, present in more than half of the bacterial domains, and partly substituted in most of the remaining domains. Only in the *Nakamurella multipartita* annexin, the GxGTD motif is missing altogether. The Glu residue located in the D/E turn is less conserved (see Figs. 1 and 3). The [RKH]G[DE] motif proposed to be involved in membrane interactions [10], [14], [23] is found only in two bacterial annexins, from *Haliangium ochraceum* DSM and from *Burkholderiales* bacterium JOSHI_001.

Interestingly, the tryptophan residue within the calcium-binding motif GWGTD that is strongly conserved in plant annexins and proposed to have an important role in membrane binding or annexin oligomerisation [24], is present in several (nine) bacterial annexin domains, and in most of the remaining domains it is replaced by a hydrophobic residue (Phe, Ile, Val or Met). This suggests the role of this residue is indeed conserved between eukaryotes and bacteria.

Another conserved tryptophan residue, reminiscent of the Trp at the C-terminus of plant annexin domain I [10], is found at the C-termini of some bacterial annexin domains; however, its role in bacterial proteins is not clear (see the alignment in Fig. S4). Finally, in contrast to some eukaryotic annexin domains, bacterial annexins contain almost no cysteine residues.

A cautionary note is needed here. Since the search for bacterial annexin homologues started with a set of ‘classical’ annexin domain sequences from the Pfam database, the fact that the homologues found by us predominantly possess the typical GxGTD/E motif, does not exclude the possibility that more diverged alternative motifs may exist in other, yet undiscovered annexin homologues.

### Phylogenetic spread of bacterial annexins and lifestyles of the host organisms

Bacterial annexins are found in just a few bacterial species spread throughout the bacterial tree of life (See Table 1). Most annexin-bearing species come from the *Bacteroidetes/Chlorobi* phylum: five from the *Cytophagia* class, *Cytophaga hutchinsonii*, *Microscilla marina*, *Fibrisoma limi*, *Fulvivirga imtechensis* AK7 and *Spirosoma linguale*, four from the *Flavobacteria* class, *Capnocytophaga gingivalis*, *Kordia algicida*, *Aquimarina agarilytica* ZC1 and *Flavobacteriaceae* bacterium HQM9, and one from the *Bacteroidia* class, *Marinilabilia salmonicolor* JCM 21150. Then, one species belongs to the *Gemmatimonadetes* phylum (*Gemmatimonas aurantiaca*), one to beta-*Proteobacteria* (*Burkholderiales* bacterium JOSHI_001) and three to delta-*Proteobacteria*, *Myxococcales* (*Haliangium ochraceum*, *Corallococcus coralloides*, *Myxococcus stipitatus* DSM 14675). Annexin genes were also found in *Actinobacteria*, *Rhodococcus imtechensis* and *Nakamurella multipartita* DSM 44233. Of note, among the taxons that contain annexin-possessing species, there are several Gram-negative phyla (*Bacteroidetes*/*Chlorobi*, *Gemmatimonadetes Proteobacteria*) and the Gram-positive phylum *Actinobacteria*. Thus, these phyla are not part of a single “superphylum”.

**Table 1:** Characteristics of bacterial strains possessing annexin genes.

Organism, reference	Motility	Oxygen requirement.	Habitat	Temperature range	Taxon
*Aquimarina agarilytica* ZC1, [75]		Aerobic	Marine, red algae		*Bacteroidetes/Chlorobi; Bacteroidetes; Flavobacteria*
*Burkholderiales bacterium* JOSHI_001, [76]	Yes		Freshwater	Mesophile	*Proteobacteria; Beta-proteobacteria; Burkholderiales*
*Capnocytophaga gingivalis* ATCC 33624, [31], [77]			Host – human	Mesophile	*Bacteroidetes/Chlorobi; Bacteroidetes; Flavobacteria;*
*Corallococcus coralloides* DSM 2259, [78]	yes		Soil	Mesophile	*Proteobacteria; Delta-proteobacteria; Myxococcales*
*Cytophaga hutchinsonii* ATCC 33406, [79]	Yes	Aerobic	Marine	Mesophilic	*Bacteroidetes/Chlorobi; Bacteroidetes; Cytophagia;*
*Fibrisoma limi* BUZ 3, [80]	No	Aerobic	Soil		*Bacteroidetes/Chlorobi; Bacteroidetes; Cytophagia*
*Flavobacteriaceae bacterium* HQM9, [81]		Aerobic	Marine, red algae		*Bacteroidetes/Chlorobi; Bacteroidetes; Flavobacteria;*
*Fulvivirga imtechensis* AK7, [82]	No	Obligate aerobe	Marine		*Bacteroidetes/Chlorobi; Bacteroidetes; Cytophagia;*
*Gemmatimonas aurantiaca* T-27, [83]	Yes	Aerobic	Marine	Mesophilic	*Gemmatimonadetes; Gemmatimonadetes (class); Gemmatimonadales;*
*Haliangium ochraceum* DSM 14365, [39]	Yes	Aerobic	Aquatic	Mesophilic	*Proteobacteria; Delta-proteobacteria; Myxococcales;*
*Kordia algicida* OT-1, [84]	No	Aerobic	Marine, red algae	Mesophilic	*Bacteroidetes/Chlorobi; Bacteroidetes; Flavobacteria;*
*Marinilabilia salmonicolor* JCM 21150, [85]			marine mud		*Bacteroidetes/Chlorobi; Bacteroidetes; Bacteroidia;*
*Microscilla marina* ATCC 23134, [86]	Yes	Aerobic	Aquatic	Mesophilic	*Bacteroidetes/Chlorobi; Bacteroidetes; Cytophagia*
*Myxococcus stipitatus* DSM 14675, [32]					*Proteobacteria; Delta-proteobacteria; Myxococcales*
*Nakamurella multipartita* DSM 44233, [87]	No	Aerobic	Waste-water, Sludge	mesophile	*Actinobacteria; Actinobacteridae; Actinomycetales; Frankineae;*
*Rhodococcus imtechensis* RKJ300, [88]	no	Aerobic	soil		*Actinobacteria; Actinobacteridae; Actinomycetales; Corynebacterineae;*
*Spirosoma linguale* DSM 74, [89]		Aerobic	Marine/Soil		*Bacteroidetes/Chlorobi; Bacteroidetes; Cytophagia*

Organism information.

Of the seventeen annexin-bearing species, twelve species are aquatic, mostly marine, while one species was isolated from waste-water treatment plant (see Table 1). Three annexin-possessing bacteria are soil bacteria. One of the annexin-bearing strains is a human pathogen, causing periodontitis (*C. gingivalis*). Most of the seventeen bacterial strains are aerobic and mesophilic. Although there is no apparent strict rule regarding the preferred habitat and lifestyle for the annexin-bearing bacteria, notably, majority of them live in an aquatic milieu where high frequency of horizontal gene transfer (HGT) has been reported [25].

### Relationships between bacterial and eukaryotic annexins

For elucidation of the evolutionary relationships between bacterial and eukaryotic annexin domains, building of a reliable phylogenetic tree is not straightforward. As a primary approach, we used an alternative, albeit less strict solution. The approximate topology of sequence similarity networks can be visualized by CLANS, a graph approach utilising sets of pairwise sequence similarities [26]. In order to illustrate the sequence similarities of the bacterial annexin domains to the eukaryotic annexins, we applied the CLANS clustering algorithm to a large set of representative eukaryotic annexin sequences identified using the Pfam database, augmented by the bacterial annexin domains identified in this work (see Methods).

**Figure 4:**
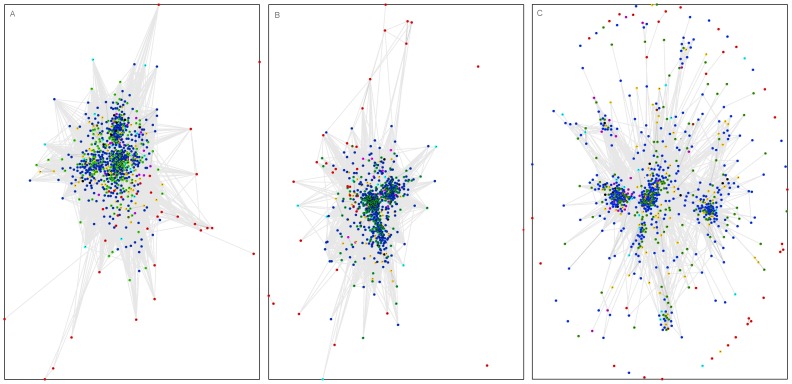
CLANS graph – sequence similarity-based clustering of bacterial annexins and known eukaryotic annexins. A) very relaxed sequence similarity threshold, B) relaxed sequence similarity threshold, C) strict sequence similarity threshold. Symbols colouring by taxonomy: red – Bacteria, blue - Metazoa, orange - Fungi, cyan - other Opisthokonts, green - plants, magenta - Stramenopiles, brown - Excavata, black - Amoebozoa. 774 representative sequences included The P-value sequence similarity thresholds used for graph building: A) 0.1, B) 1e-3, C) 1e-7.

In the CLANS clustering, using various sequence similarity significance thresholds, one obtains a consistent picture in which bacterial annexin domains locate away from the known annexins (See Fig. 4). Notably, bacterial annexin domains do not cluster with the atypical giardin domains from the protozoan *Giardia* nor with the annexin domains from other unicellular eukaryotes. Some bacterial annexins apparently show no similarity to eukaryotic annexins or to each other in the CLANS graph. This is only because the graph utilises BLAST derived similarity relationships. The original discovery of bacterial annexin-like domains was done using more sensitive tools (e.g. HHpred and FFAS, see Methods). Nevertheless, the clustering picture reflects the large diversity of bacterial annexins and suggests their ancestral origin. Yet, the clustering analysis does not suffice to clearly resolve the origins of bacterial and eukaryotic annexins.

In a phylogenetic tree of bacterial annexins (See Fig. S1), some domains within one species (*Haliangium ochraceum* and *Flavobacteriae*, e.g. *Kordia algicida*) group together which could suggest that these bacterial strains probably acquired single annexin domains, and then duplication occurred within some species. The *Flavobacteriae* branch is split into two sub-branches indicating a common origin of flavobacterial annexins and the presence of a pair of single-domain annexins in the flavobacterial ancestor.

Strikingly, in some cases, different annexin domains from a single bacterial protein occur in two separate main branches of the tree (Fig. S1, e.g. annexin domains from *Burkholderiales* bacterium, *G. aurantiaca*, *C. coralloides* and *M. stipitatus*. Such a pattern suggests that the individual multiple annexin domains within some bacterial proteins did not arise by gene duplication in the particular strains. Instead, this pattern suggests that a multi-domain annexin protein existed already at some earlier time point in bacteria and was later transferred to various phyla and individual strains by vertical evolution or, possibly, by horizontal gene transfer.

**Figure 5:**
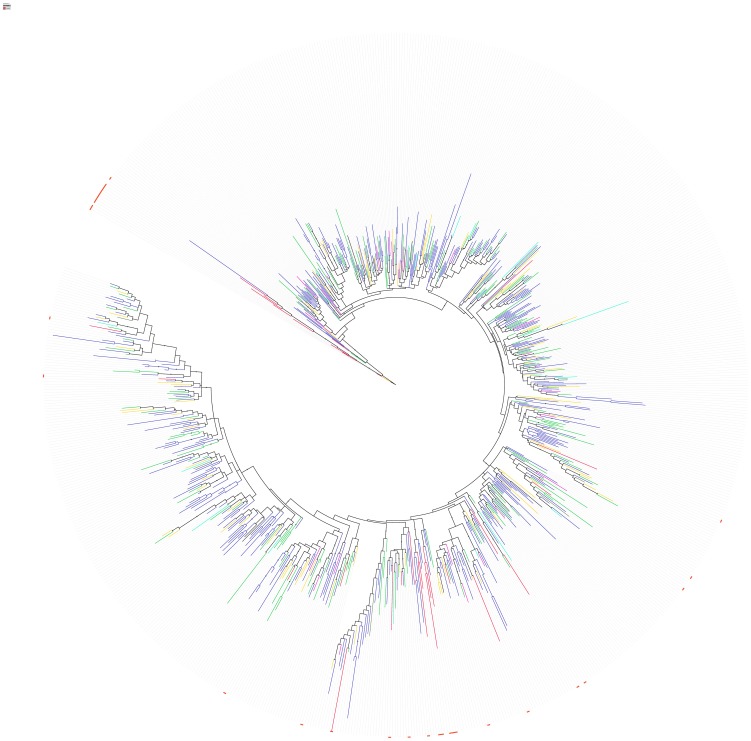
Phylogenetic tree of 774 representative annexin domain sequences. Multiple sequence alignment built using the MAFFT program. Phylogeny built using the PhyML algorithm. Branch colouring by taxonomy, as in Fig. 4. Approximate bootstrap values obtained using the aLRT test. Branches with bootstrap values below 0.75 collapsed, dots on branches indicate bootstrap values above 0.9.

As said above, phylogenetic trees including eukaryotic and bacterial annexin domains are not robust and sensitive to details of the underlying sequence alignments. In the case of annexin domains, the sequences are relatively short and very diverse, which augments difficulties. Nevertheless, we attempted to build such a tree (see Fig. 5 and Fig. S2). Despite the approximate bootstrap values that support many branches of the sample tree, the tree is not robust with respect to changing sequence alignment methods and alignment curation approaches (not shown). Thus, one can have rather limited confidence in the detailed topology of the phylogenetic tree. A general feature of the tree shown and alternative trees tested is that bacterial annexin homologues form 1–3 clades interspersed among the eukaryotic annexin branches, often with Metazoan annexin repeats as the nearest neighbours in the trees. Also, some annexin sequences group close to the root of the tree. Neither the sample tree nor its alternatives can decisively resolve the question on the origin of bacterial annexins, however the tree suggests their ancient origin.

### Hypotheses regarding the biological processes that bacterial annexins may be involved in

Several hypotheses as to specific roles of bacterial annexins may be proposed. Analysis of the genomic neighbourhoods of bacterial annexins gives no general clue to their function, however in *Kordia algicida*, metalloproteases are consistently present in the vicinity of annexins. Considering the putative metalloprotease domains (DUF4157, and Peptidase_M90) present in *Haliangium* annexin proteins, regulation of proteolysis may be a function of at least some bacterial annexins. One specific hypothesis would be annexin-mediated regulation of protease excretion in *K. algicida*, where protease excretion has been shown to be required for algicidal action of the bacterium on diatoms [27]. Likewise, in humans, annexins have been shown to regulate metalloprotease-mediated processes, namely shedding of the signalling molecule proamphiregulin [28].

Another hypothesis could be related to a role of bacterial annexins in the regulation of biofilm formation [29]. The presence in biofilms has been described for a number of annexin-bearing strains. For example, dental biofilm has been shown to include *Capnocytophaga gingivalis* [30], [31] and freshwater stream biofilm has been shown to include *Burkholderiales* bacterium JOSHI_001.

Some bacteria are known to form multicellular, spore-filled fruiting bodies in processes analogous to multicellular morphogenesis and cellular differentiation in eukaryotes. It may be hypothesized that such bacteria might be more likely than others to use eukaryotic-like proteins to assist in this kind of processes. Among the annexin-possessing bacteria, fruiting bodies are formed by some *Myxococcales*, *Corallococcus coralloides* DSM 2259, *Haliangium ochraceum*, *Myxococcus stipitatus* [32].

In *Burkholderiales* bacterium JOSHI_001, the annexin molecule has a peculiar domain architecture. It is predicted to be cytosolic, membrane anchored, with at least three annexin domains proximal to the membrane anchor, and a Colicin D domain, with a sequence significantly similar to the Colicin D toxin which is acting as specific ribonuclease against tRNA(Arg) [33]. Towards the N-terminus from the Colicin D domain lies a Pyocin_S domain (PF06958), found at the N-termini of some colicin molecules and acting as a translocation domain [34]. This suggests that at least in this case annexin domains may be involved in a bactericidal/infectious process.

In *Microscilla marina* ATCC 23134, the annexin domain is fused to a lipase type 3 domain (Pfam PF01764). The lipase active site Ser, His and Asp residues are perfectly conserved. Thus, in *M. marina*, the annexin domain may be involved in regulating a lipase activity. This is not surprising given the known involvement of eukaryotic annexins in lipid signalling [35] The *M. marina* annexin possesses also a novel toxin domain, Ntox_3 (Toxin_58) predicted to act as RNAse [36]. Thus, annexin and lipase domains, probably by membrane binding and/or insertion activity, may mediate actions of the toxin domain and assist in its delivery to the attacked cell.

Interestingly, some *Haliangium ochraceum* annexins (e.g. gi|262077350, see Fig. 2) possess the same Ntox_3 toxin domain in addition to a metalloprotease domain. In these proteins, the metalloprotease and annexin domains probably act in toxin processing and delivery, respectively. In another *H. ochraceum* annexin (e.g. gi|262194969) that lacks the Ntox_3 domain, a second metalloprotease domain (Peptidase_M90, similar to anthrax lethal factor metallopeptidase) located at the C-terminus may act as toxin.

Some bacterial annexin proteins have one or more predicted transmembrane regions (see Fig. 2). This supports the idea that, like their eukaryotic annexin homologues, bacterial annexins are performing their functions by and while interacting with cell membranes. Finally, eukaryotic annexins are known to be interacting with actin and regulate the actin cytoskeleton function with implications for cell motility, cell polarity, endocytosis and cytokinesis [6], [37], [38]. Interestingly, one of the annexin-bearing bacteria, *Haliangium ochraceum*, has been reported to possess the first actin homologue identified in a bacterium [39]. This invites speculation that some bacterial annexins may regulate bacterial cytoskeleton.

## Conclusions

We have surveyed putative bacterial annexins and found only thirty four of them in seventeen bacterial species coming from as many as four phyla: *Bacteroidetes*/*Chlorobi*, *Gemmatimonadetes, Proteobacteria* (beta- and delta-), and *Actinobacteria*.

In summary, three evolutionary scenarios could explain the observed occurrence of annexin domains in bacterial proteins:

1. annexin domain(s) were present in the Last Universal Common Ancestor (LUCA) and were subsequently lost in many lineages, including most bacterial taxa. Alternatively, in many of the apparently ‘annexin-free’ lineages the annexin homologues might have diverged beyond recognition by the currently available sequence comparison methods.
2. annexin domain(s) originate from some specific bacterial phylum and were transferred to early eukaryotes (and possibly some other bacterial taxa) by horizontal gene transfer.
3. annexin domain(s) originate from an early eukaryotic ancestor, and were transferred to bacteria by one or more events of horizontal gene transfer.
4. similarity between eukaryotic and bacterial annexins could be a result of convergent evolution and there could be no homology between them. However, the conservation of typical annexin sequence motifs and overall sequence similarity makes this possibility seem unlikely.

The scenario number 1 has to account for the paucity and scarce phylogenetic distribution of annexins in bacteria. In the *Bacteroidetes/Chlorobi* phylum, there are 10 species possessing annexins while 88 complete genomes for this phylum are listed at ncbi.nlm.nih.gov. For beta-*Proteobacteria*, there is one annexin-bearing species and 216 genomes known, for delta-*Proteobacteria* the numbers are 3 vs 48, for *Actinobacteria* 2 vs 144 and finally for *Gemmatimonadetes*, 1 vs 1. Thus, this scenario would implicate massive annexin gene losses in many lineages or very divergent evolution leading to the oversight of more divergent annexins in the current analysis. The gene losses could be related to lack of functional requirement for annexins in many bacterial lineages.

On the other hand, horizontal gene transfer (HGT) of annexins between eukaryotes and bacteria and within bacteria would be probably the most parsimonious explanation of the observed presence of rare bacterial homologues. The phenomenon of HGT between multicellular eukaryotes and bacteria is gaining in recognition since the landmark studies of gene exchange between intracellular bacterial parasites and their insect hosts [40]–[44]. These discoveries, albeit fascinating, involved obvious proximity between the genetic material of the bacterial pathogen and the Metazoan host. In some other cases reported, the very remote similarity between bacterial and Metazoan sequences precluded accurate delineation of the possible path or location of the hypothetical HGT event. For example, we have described recently bioinformatics evidence pointing at likely cases of horizontal gene transfer occurring between bacteria and Metazoans that involved human disease-related proteins, putative peroxiredoxins and metalloproteases [45], [46]. Various other cases of horizontal gene transfer between bacteria or unicellular eukaryotes and multicellular eukaryotes have been also reported by several groups recently, based on diverse arguments, including phylogenetic analyses and three dimensional structure determination [47]–[51]. Often, in cases of singular, or extremely patchy phylogenetic distribution of proteins, horizontal gene transfer has been argued for as the most parsimonious explanation of the data [46], [49]. A bioinformatics method has been designed for prediction of horizontal gene transfer by discovering phylogenetically atypical genes on a genome-wide basis [52].

Although the HGT scenario could be most parsimonious one, no strong evidence for it can be seen in the sequence data; in particular there are no strong similarities observed between annexins from bacteria and eukaryotes as would be expected in case of xenologues. Assuming the HGT scenario holds, we could not determine whether eukaryote-bacteria HGT occurred once or several times. Also, several events of bacteria-to-bacteria HGT would be required to account for the current annexin distribution.

Nevertheless, notwithstanding the peculiar phylogenetic distribution of annexins in bacteria, the most likely evolutionary scenario that the data suggest is the following: bacterial annexins descend from ancestral molecules that were probably already multidomain proteins, and were present early in bacterial evolution, or, possibly, in the last universal common ancestor, LUCA. This view is supported by the large variation of bacterial annexin sequences and by the rich repertoire of structural domain combinations observed alongside annexin domains in bacterial proteins. However, until more distant homologues of annexins are found in prokaryotes and possibly viruses, until more genomes of unicellular eukaryotes from different taxonomic lineages are charted, the origin of the bacterial annexins cannot be proven beyond any doubt. Further, the precise delineation biological roles of annexins in bacteria awaits detailed experimental studies.

## Methods

### Annexin sequence survey

For identification of remote annexin homologues in bacteria, the Jackhmmer tool from the HMMER suite was used [53], using the Pfam seed alignment for the Annexin domain (PF00191) as query against the Uniprot database (as of January 2013). The search converged in seven iterations. Additionally, the alternative HHsenser tool [18] that adopts a somewhat more greedy approach, was used with the same Pfam seed alignment of the Annexin domain (PF00191) as query on the NCBI nr database.

The Jackhmmer and HHsenser hits were confirmed using HMMER3 [18] on the Pfam database [54]. Those that could not be thus confirmed, were double-checked using the FFAS03 method [55], that uses sequence profile-to-profile comparison and the HHpred algorithm [56] that employs HMM-to-HMM comparison.

Additionally, the curated RefSeq database at NCBI [19] was queried on January 19, 2013, for bacterial proteins that are annotated in any text field as “annexin”, “annexin-like” or “putative annexin”. Also these proteins were double-checked using Pfam HMM, FFAS03 and HHpred. Finally, the homologues of the bacterial annexin domains found were explored by analysis of PSI-Blast search results [57].

For additional domain assignments in the bacterial annexin proteins, the HMMER3 [58] on the Pfam database as of March 2013 was used.

### Bacterial annexin sequence variability and motifs

Multiple sequence alignment of bacterial annexin domains was built using the Promals3D and MUSCLE programs [59], [60] and manually curated in the vicinity of the GxGTD motif. The Jpred and PsiPred servers were used to predict the secondary structures [61], [62]. The secondary structure prediction shown in Fig. 3 (Jnet method) uses the information contained in the whole sequence alignment as opposed to single sequences. It also provides confidence of secondary structure prediction at each position. Phylogenetic tree of bacterial annexin domains was built using the PhyML maximum likelihood algorithm as implemented on the phylogeny.fr server [63]. The close homologues were omitted from the alignment by filtering out redundancy at 70% sequence identity.

For sequence logos, the WebLogo tool was used [64]. The sequences represented in the logo are obtained from the alignment shown in Fig. 3. For presentation of multiple sequence alignments, the JalView and BioEdit software were used [65], [66]. Transmembrane region predictions were achieved by the TMHMM and Phobius servers [67], [68].

### Phylogeny of bacterial annexins and relationships to eukaryotic homologues

For survey of sequence similarities among annexin domains, a set of representative annexin domains was built as follows. All the occurrences of the Pfam annexin domain from the Uniprot database (Pfam version 27.0, March 2013) with the addition of bacterial annexin domains identified in this study were clustered at 60% sequence identity threshold using CD-HIT [69]. Multiple sequence alignment was performed using the MAFFT program [70]. The alignment was curated using the Trimal program [71] on the Phylemon2 web server at http://phylemon.bioinfo.cipf.es by removing columns with more than 50% of gaps. Then, sequences containing 12 gaps or more were removed. The set thus built consisted of 774 annexin domain sequences. The CLANS algorithm [26] was run on the representative annexin domain set of sequences, using BLAST with the BLOSUM45 substitution matrix. For the CLANS graphs, sequence similarity relations with significance of P-values below 0.1, 0.001 and 1E-7 were considered, as indicated in Fig. 4.

For constructing a phylogenetic tree, the MAFFT alignment of representative annexin domain sequences was used, the Prottest server was used to select the most appropriate model of protein evolution, and the LG+G model was found to suit the data best. Thus, the phylogenetic tree was built using the PhyML algorithm (on the http://www.atgc-montpellier.fr server).

### Properties of annexin-possessing bacteria

Information on the environments and lifestyles of annexin-bearing bacteria were collected from the Genome database at NCBI [72], from the GOLD database [73], and from literature (see Table 1). Genomic neighbourhoods of annexin-possessing bacteria were analysed in the Microbes Online portal [74].

## Supporting Information

Figure S1 **Phylogenetic tree (PhyML) of bacterial annexin domains.** Branches with approximate bootstrap values (aLRT) above 0.9. marked with dots. Branch colouring by taxonomy: yellow: *Bacteroidetes/Chlorobi*, green: *Gemmatimonadetes*, blue: beta-*Proteobacteria*, red: delta*-Proteobacteria*, grey: *Actinobacteria*.
(PNG)

Figure S2 **Expanded version of Figure 5.** Phylogenetic tree of 774 representative annexin domain sequences, numbers correspond to identifiers shown in the list.
(PDF)

Figure S3 **HHpred results of sequence similarity analysis of wider regions of bacterial annexin proteins, showing weak sequence similarities to annexin tetrads.**
(PDF)

Figure S4 **Multiple sequence alignment (Promals3D) of bacterial annexin domains.** This is full, unedited version of the alignment shown in Fig. 4.
(PNG)

Table S1 **List of bacterial annexin domains.** Protein identifiers, location of the domains within the protein sequence, and their sequence similarity to known eukaryotic annexins.
(XLS)
