# Supplementary figures and images for: Bioinformatics Analysis of Bacterial Annexins – Putative Ancestral Relatives of Eukaryotic Annexins
Source: PLoS One. 2014 Jan 16;9(1):e85428. doi: 10.1371/journal.pone.0085428 (PMC3894181; doi:10.1371/journal.pone.0085428)

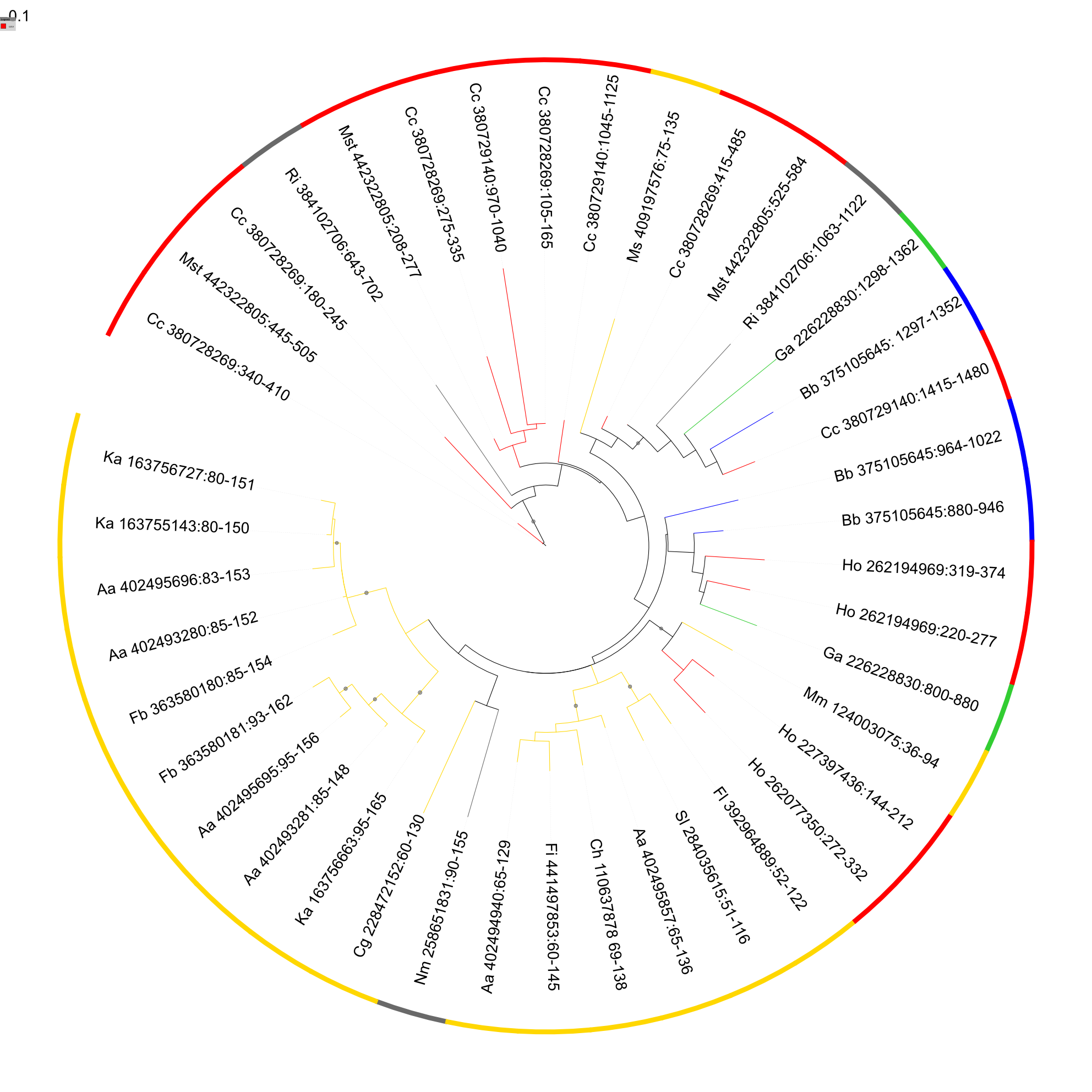

Supplement: Figure S1 — Phylogenetic tree (PhyML) of bacterial annexin domains. Branches with approximate bootstrap values (aLRT) above 0.9. marked with dots. Branch colouring by taxonomy: yellow: Bacteroidetes/Chlorobi, green: Gemmatimonadetes, blue: beta-Proteobacteria, red: delta-Proteobacteria, grey: Actinobacteria. (PNG) [file pone.0085428.s001.png]

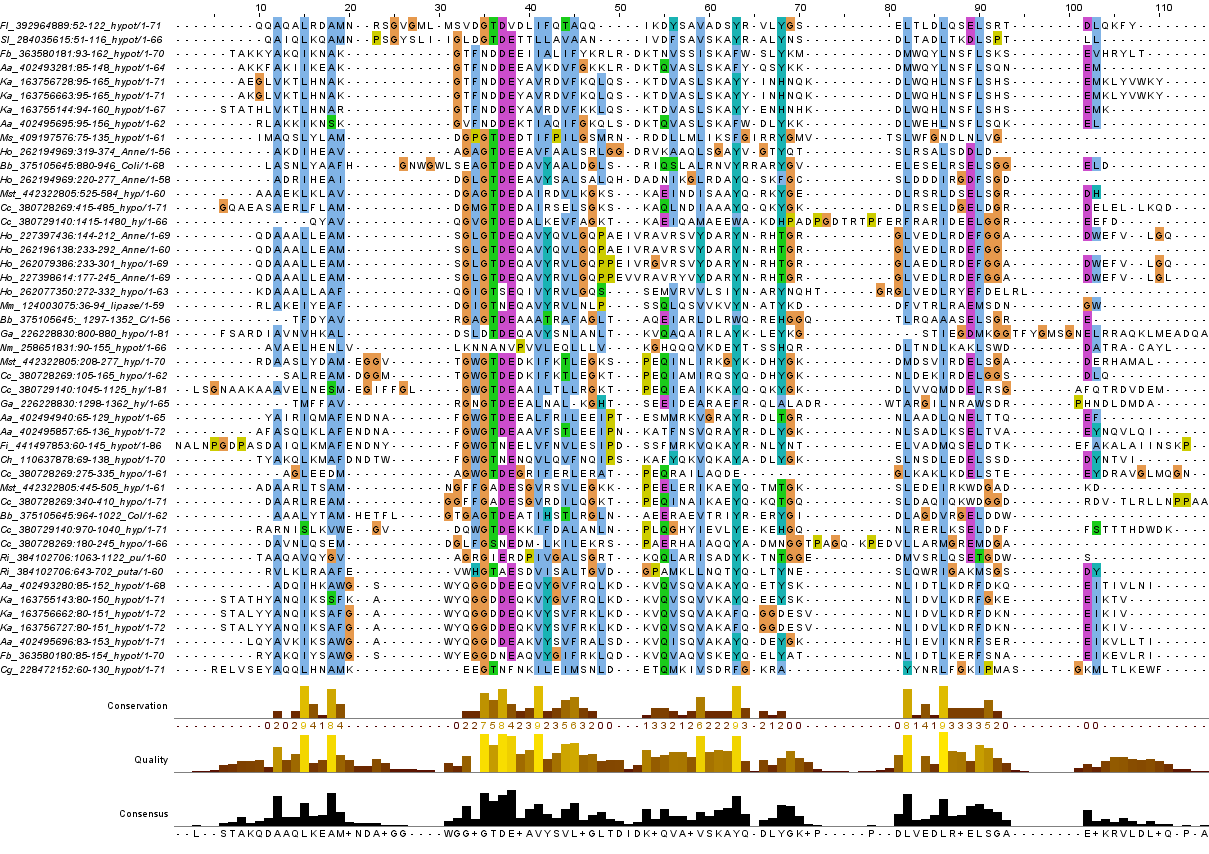

Supplement: Figure S4 — Multiple sequence alignment (Promals3D) of bacterial annexin domains. This is full, unedited version of the alignment shown in Fig. 4. (PNG) [file pone.0085428.s004.png]
