# Supplementary material for: Bioinformatics Analysis of Bacterial Annexins – Putative Ancestral Relatives of Eukaryotic Annexins
Source: PLoS One. 2014 Jan 16;9(1):e85428. doi: 10.1371/journal.pone.0085428 (PMC3894181; doi:10.1371/journal.pone.0085428)
Supplement: Figure S2 — Expanded version of Figure 5. Phylogenetic tree of 774 representative annexin domain sequences, numbers correspond to identifiers shown in the list. (PDF) [file pone.0085428.s002.pdf]

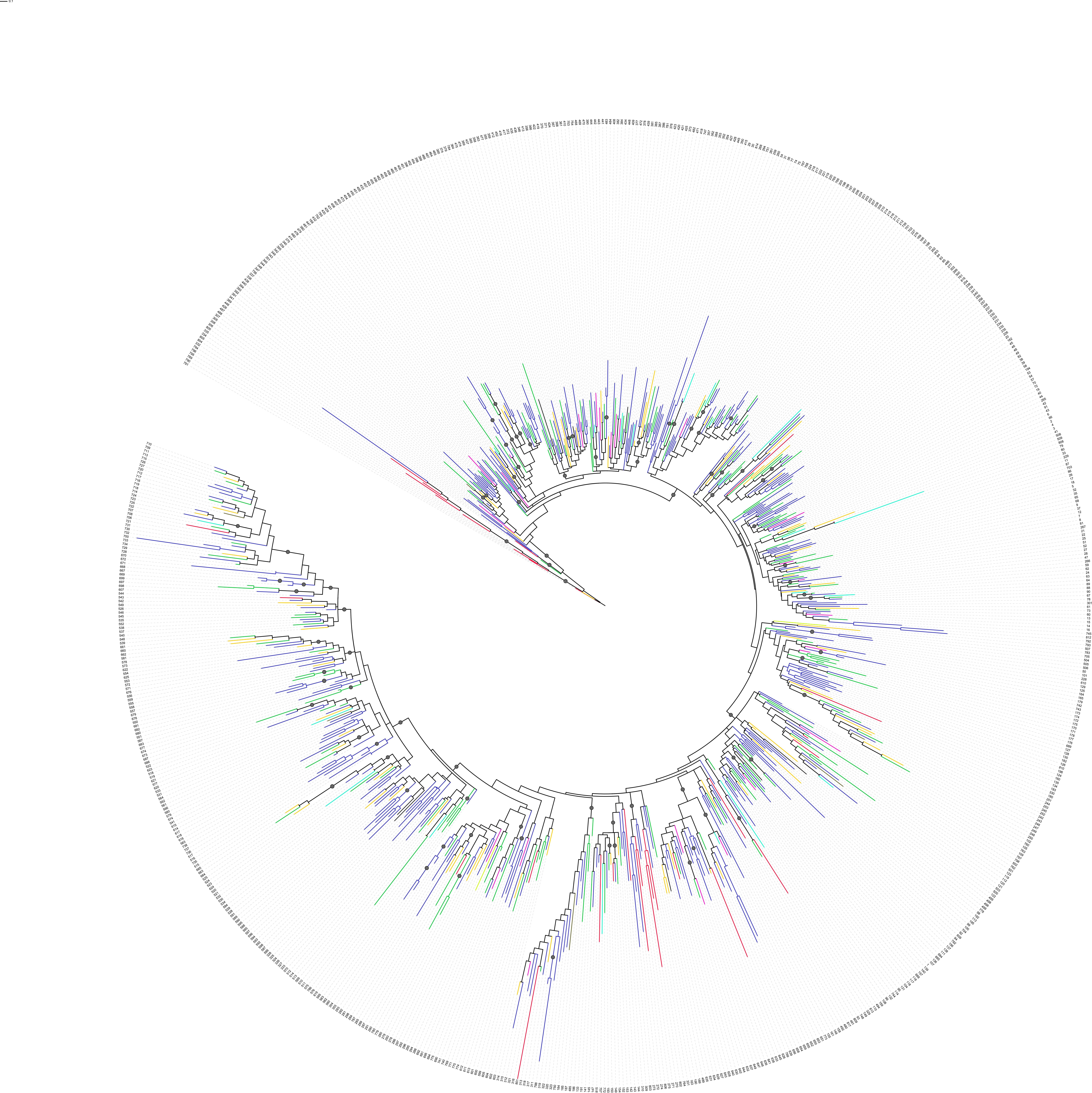

1	Amoebozoa_F0YFB9_AURAN/158-223
2	fungi_A1C4F1_ASPCL/231-296
3	plant_A5AJ78_VITVI/169-235
4	plant_A9RZF7_PHYPA/169-235
5	plant_A5BTZ8_VITVI/163-228
6	plant_E0ZCA8_PICSI/63-126
7	Metazoa_H2N0L3_ORYLA/7-72
8	Metazoa_Q5U369_DANRE/269-334
9	Metazoa_F6T207_ORNAN/118-183
11	Metazoa_Q6GNT0_ENLA/253-318
12	fungi_A1DHU3_NEOFI/301-367
13	Metazoa_C1BW6_ESOLU/193-259
14	Stramenopiles_B8CCQ1_THAPS/96-161
15	Metazoa_A6RL81_BOTFB/388-444
16	Metazoa_G1KMZ9_ANOCA/171-237
17	plant_F2E4W7_HORVD/15-75
18	Metazoa_G3WNV2_SARHA/20-85
19	Metazoa_F6UBG2_CALJA/196-264
20	plant_A5B479_VITVI/245-310
21	Metazoa_A7T6G0_NEMVE/1-57
22	plant_C1E164_MICSR/438-503
23	Metazoa_G3N8C5_GASAC/269-334
24	Other_O_F2UJT2_SALS5/272-337
25	Metazoa_A8NZK8_BRUMA/155-207
26	Metazoa_C3ZV5_BRAFL/820-872
27	plant_B9GM28_POPTR/15-80
28	Metazoa_H3EJI1_PRIPA/81-142
29	Metazoa_E1GHD6_LOALO/1-55
30	Metazoa_F4W699_ACREC/97-162
31	Metazoa_E9G1C8_DAPPU/27-92
32	plant_I1I762_BRADI/15-77
33	Metazoa_E1GHD6_LOALO/73-139
34	plant_P93158_GOSHI/13-79
35	Stramenopiles_B8CCQ1_THAPS/23-89
36	Metazoa_C1BMC5_OSMMO/173-239
37	Metazoa_C1BZS7_ESOLU/94-159
38	Metazoa_B4L73_DROMO/19-84
39	plant_B3TLY9_ELAGV/170-235
40	plant_B4FJC1_MAIZE/170-240
41	Other_O_E9C3C5_CAPO3/984-1050
42	Metazoa_B51W5_SALSA/408-474
43	Metazoa_H2LY47_ORYLA/111-176
44	Metazoa_H0YRC2_TAEGU/358-423
45	Metazoa_A2BHN0_DANRE/111-176
46	Metazoa_A7SW86_NEMVE/184-250
47	fungi_F7W8H2_SORMK/405-471
48	Metazoa_E2RCI8_CANFA/254-319
49	Metazoa_Q803A1_DANRE/89-154
50	plant_B9I5V5_POPTR/248-314
51	Stramenopiles_G4Z1N4_PHYSP/25-91
52	Metazoa_G4VI66_SCHMA/401-467
53	Metazoa_C1BZR8_ESOLU/20-85
54	Metazoa_G1MU27_MELGA/16-82
57	Metazoa_Q7ZW0_ENLA/266-331
58	Metazoa_H3FTB2_PRIPA/227-281
59	Metazoa_F6W0Q1_MONDO/245-310
60	plant_O82090_GOSHI/170-235
61	Metazoa_E1BW1_CHICK/437-502
62	fungi_G9NH54_HYPAI/148-213
63	plants_D8LG01_ECTSI/443-507
64	Metazoa_Q3US43_MOUSE/46-111
65	Metazoa_A5LHA3_LITCT/270-335
66	fungi_F0PB2_GROCL/165-230
67	Metazoa_ANA5_CHICK/174-240
68	plant_C0PQS6_PICSI/171-228
70	plant_B9HVR2_POPTR/87-152
72	plant_D2D2Z9_GOSHI/86-151
73	fungi_C0ND81_AJECG/181-246
401	Metazoa_Q6DKC4_ENLA/110-175
402	Metazoa_H3BCF7_LATCH/91-156
403	Metazoa_A5LHA5_LITCT/250-315
404	Stramenopiles_G4Z1N4_PHYSP/272-336
405	plant_B9HVR2_POPTR/170-236
406	fungi_F7W8H2_SORMK/327-394
407	Metazoa_C1BIH9_OSMMO/89-154
408	fungi_E7A392_SPORE/127-191
409	Stramenopiles_H3H2V8_PHYRM/97-163
410	plant_D3GC08_9ROSI/14-79
412	fungi_B6GYL8_PENCW/333-388
413	Metazoa_G4VI63_SCHMA/343-409
414	Metazoa_E0VHI3_PEDHC/70-135
415	Metazoa_Q7QG24_ANOGA/252-317
416	plant_E5GCK3_CUCME/87-152
417	Metazoa_G6D7C8_DANPL/176-241
418	fungi_H1VE74_COLHI/316-381
419	Stramenopiles_B5Y5D2_PHATC/262-324
420	Metazoa_E9J8L4_SOLIN/97-162
421	Other_O_A9V2E0_MONBE/829-895
422	Metazoa_D6WZQ2_TRICA/164-229
423	Metazoa_F1L2C7_ASCSU/92-157
424	Metazoa_C1BKB4_OSMMO/37-102
425	Metazoa_G0MCK6_CAEBE/248-313
426	fungi_Q0CEU8_ASPTN/288-354
427	plant_Q9ZR53_MEDSA/87-151
428	Metazoa_G5EEA8_CAEEL/248-313
429	Other_O_A9UPZ0_MONBE/252-317
430	Metazoa_H3B470_LATCH/256-321
431	plant_B9I4U4_POPTR/89-152
432	Metazoa_D6WZQ1_TRICA/83-148
433	Amoebozoa_ANA7_DICDI/319-386
434	Metazoa_Q5DHI5_SCHJA/195-262
435	Metazoa_Q17A50_AEDAE/93-158
436	Metazoa_Q5D8M0_SCHJA/102-168
437	plant_I1HJW8_BRADI/225-289
438	fungi_A2QCY7_ASPNC/148-213
439	Other_O_A9VC1_MONBE/664-730
440	plant_A5B7L0_VITVI/176-221
441	plant_I1JWG5_SOYBN/221-286
442	fungi_B2VUR2_PYRTR/397-464
443	Metazoa_F6WI47_ORNAN/91-156
444	Stramenopiles_D0NJ65_PHYIT/184-250
445	Metazoa_F6T207_ORNAN/277-342
446	Stramenopiles_F0WGW3_9STRA/514-580
447	Stramenopiles_D0NUM8_PHYIT/183-249
448	plant_G1ETN3_NELNU/244-309
449	Metazoa_G5DZW5_9PIPI/170-236
450	Metazoa_G1Q8S6_MYOLU/402-450
451	Metazoa_G1P7U7_MYOLU/60-126
452	Metazoa_B7QCG4_IOSC/101-146
453	plant_A5B479_VITVI/87-152
454	plant_A9TFB3_PHYPA/245-310
455	plant_AND7_ARATH/245-310
456	Metazoa_F7HMN3_CALJA/21-80
457	Metazoa_D6WZQ1_TRICA/777-842
458	fungi_B8M4F7_TALSN/405-470
459	Metazoa_F7BN50_ENTR/178-244
460	Metazoa_E3MY10_CAERE/102-167
461	Metazoa_H3B3V1_LATCH/202-268
462	fungi_C5FNC5_ARTOC/403-470
463	Metazoa_H3CR25_TETNG/18-83
464	Metazoa_E3MY10_CAERE/32-95
465	Metazoa_Q6P603_DANRE/107-172
466	Metazoa_B4M9Y2_DROVI/251-316
467	Metazoa_C0HA2_SALSA/301-366
468	Other_O_A9VD41_MONBE/154-190
469	Metazoa_E39D4_ANODA/102-167

74 Metazoa_C3ZI54_BRAFL/47-92
75 Stramenopiles_G5AHT5_PHYSP/899-965
77 Metazoa_H3HWB9_STRPU/160-225
78 Metazoa_H2S105_TAKRU/275-340
79 plant_C5NL1_SORBI/252-319
80 Metazoa_G3VRW3_SARHA/89-154
81 plant_AN4_FRAAN/169-234
82 Metazoa_E1FYF7_LOALO/253-318
83 plant_B7U9S0_CARAS/185-250
84 Metazoa_G3WNV2_SARHA/251-316
85 plant_I1LLB8_SOYBN/274-330
86 fungi_H1VDA4_COLHI/658-694
87 Metazoa_G3PY92_GASAC/254-319
88 plant_A2YVD9_ORYSI/171-237
89 Other_O_F2UBR9_SALS5/86-152
90 Metazoa_H2RYZ9_TAKRU/47-112
91 Metazoa_Q640T3_ENTR/93-158
92 Metazoa_A3KN40_BOVIN/97-163
93 Metazoa_A7T6G0_NEMVE/64-129
94 Metazoa_D5KU33_BOMM/270-335
95 plant_A5AJ78_VITVI/241-308
96 Metazoa_G3Q341_GASAC/248-313
98 fungi_A6R2M2_AJECN/413-480
99 Metazoa_G5BQN3_HETGA/192-258
100 Metazoa_D3ZSA0_RAT/117-182
101 Metazoa_Q66KY7_ENLA/135-200
102 Metazoa_H2VQB3_CAEJA/266-331
104 Metazoa_B2L3Y3_ENBO/1-28
105 plant_F6KLJ6_ARAHY/168-235
107 Metazoa_Q6DKC4_ENLA/269-334
108 plant_B9SNI5_RICCO/8-74
109 Metazoa_D6WDY4_TRICA/25-90
110 plant_B6U6E7_MAIZE/170-227
111 plant_A9SPZ1_PHYPA/272-337
112 plant_B4FSS8_MAIZE/174-243
113 Metazoa_F6TPA0_MONDO/165-202
114 fungi_E3QNG4_COLGM/236-301
116 plant_A9NNL2_PICSI/15-80
117 Stramenopiles_D0NJ65_PHYIT/99-164
118 Metazoa_Q804G4_DANRE/297-362
119 Metazoa_F6W0Q1_MONDO/14-79
120 fungi_F974_MYCGM/376-442
121 bacteria_Cg_228472152.30
122 Metazoa_F6VNG2_CIOIN/356-423
123 Metazoa_B0V2N5_MOUSE/46-107
124 Metazoa_A6RW25_BOTFB/235-300
125 plant_D8R2M8_SELML/87-152
126 Metazoa_E9H188_DAPPU/188-256
127 Metazoa_H2N0L3_ORYLA/164-229
128 Metazoa_G3MG82_9ACAR/15-80
129 fungi_C1GNP1_PARBA/263-328
130 Metazoa_H2ZUH7_LATCH/177-243
131 plant_A2YVD9_ORYSI/15-80
132 Metazoa_G1SKA9_RABIT/39-102
133 plant_A9SB32_PHYPA/212-279
134 Metazoa_B4GM07_DROPE/256-321
135 Metazoa_F6M44_CIOIN/22-90
136 plant_B9RGC8_RICCO/244-309
137 Metazoa_F7DNG2_HORSE/117-182
138 Metazoa_H2RWB9_TAKRU/22-91
139 plant_C6TK93_SOYBN/172-237
140 Metazoa_Q66ID8_DANRE/39-104
141 Metazoa_H2MEL1_ORYLA/278-343
142 fungi_B2WAQ5_PYRTR/601-654
143 plant_O65848_MEDTR/86-150
144 Metazoa_C3ZND4_BRAFL/94-159
145 Metazoa_F1C6Z5_PERFV/1-53
147 Metazoa_O93446_ORYLA/110-175
470 Stramenopiles_Q0ZNW4_SAPMO/259-323
471 Metazoa_E1GLF4_LOALO/38-103
472 Metazoa_Q4W6D6_9METZ/176-242
473 plants_D8LG01_ECTSI/96-162
474 Metazoa_Q4T4Q3_TETNG/240-305
475 Amoebozoa_Q54PW0_DICDI/335-395
477 Metazoa_B7QCG4_IOSC/29-94
478 plant_D6Q29_SOYBN/298-365
479 Metazoa_Q4SJ99_TETNG/224-289
480 Metazoa_G3H29_CRIGR/242-307
481 Metazoa_F1N650_BOVIN/46-111
482 Metazoa_E1G226_LOALO/185-250
483 Metazoa_G2HJV7_PANTR/167-232
484 Metazoa_G7P536_MACFA/253-318
485 plant_A9RBN7_PHYPA/86-155
486 Other_O_E9C3C5_CAPO3/615-681
487 Metazoa_F6YYM0_MONDO/45-110
488 Metazoa_H2URI3_TAKRU/248-313
489 plant_A2YAT3_ORYSI/15-80
490 plant_C6TK93_SOYBN/15-80
491 Metazoa_E9G1C8_DAPPU/99-164
492 Metazoa_G3PCE2_GASAC/38-103
494 Metazoa_ANA1_RAT/118-183
495 Ecavates_E2RTS4_GIAIC/226-283
496 Metazoa_A2BHN0_DANRE/270-335
498 Metazoa_Q5U362_RAT/18-83
499 Other_O_F2UPY8_SALS5/412-478
500 plant_Q8W4Z7_GOSHI/57-122
501 bacteria_Aa_402495696.11
502 Metazoa_H3BDR7_LATCH/192-258
503 plant_B9I4U4_POPTR/15-80
504 Metazoa_A8KBY8_DANRE/91-156
505 Metazoa_E2R0S6_CANFA/25-90
506 Metazoa_C3VEV0_SCHMA/195-262
507 human_Q5VTM3_HUMAN/162-200
508 fungi_F9QC8_MYCGM/541-590
509 Stramenopiles_G5AHT5_PHYSP/977-1040
510 fungi_C1GIN4_PARBD/330-395
511 Metazoa_ANA1_BOVIN/118-183
512 Metazoa_Q4SNZ4_TETNG/83-148
513 Metazoa_Q6Y223_PAGMA/45-111
514 Metazoa_G1KKD7_ANOCA/367-432
515 Metazoa_E0VHI3_PEDHC/301-350
516 Metazoa_E4VU6_OIKDI/184-250
517 Metazoa_F7A0T0_HORSE/47-112
518 plant_A2YAT3_ORYSI/87-154
519 plant_A5AQM9_VITVI/164-217
520 plant_A5BLT1_VITVI/169-236
521 Metazoa_E9GWA4_DAPPU/73-137
522 Metazoa_H3APK8_LATCH/277-342
523 fungi_G2WZD9_VERDV/305-370
524 Metazoa_F7FNN0_ORNAN/109-174
525 Metazoa_F1QRP0_DANRE/90-155
526 Metazoa_ANA8_MOUSE/181-247
528 Metazoa_G4VI63_SCHMA/187-252
529 Metazoa_O93447_ORYLA/362-428
530 plant_A9SB32_PHYPA/58-114
531 Metazoa_B2L3Y8_ENBO/1-27
532 fungi_G1WYE9_ARTOA/267-332
533 Ecavates_C6LZB5_GIAIB/86-146
534 Metazoa_H9G957_ANOCA/290-356
535 plant_A5BTZ8_VITVI/8-73
537 Metazoa_ANA1_RODSP/118-183
538 Metazoa_A8ZC3_CAEBR/18-81
539 fungi_Q5KH35_CRYNJ/333-397
540 plant_I1HFK2_BRADI/171-231
541 Metazoa_B5FE8_TAEGU/14-79
542 fungi_C1G8V6_PARBD/420-489

148 plant_A5B479_VITVI/15-80
149 fungi_B2B343_PODAN/248-312
150 plants_D7FJE4_ECTSI/267-332
152 Metazoa_F6PT14_HORSE/59-124
153 plant_G7LF84_MEDTR/86-151
154 bacteria_Ho_227398614.7
155 Metazoa_B1B561_SOLSE/162-227
156 Metazoa_H3IJQ8_STRPU/22-86
157 plant_E4ME0_THEHA/1-46
158 fungi_Q5MAH1_PENCH/48-112
159 Metazoa_F67L6_CALJA/214-280
160 Metazoa_B7U3W3_CTEID/92-157
161 Metazoa_F6WVJ1_ORNAN/248-312
162 Metazoa_B5RI22_SALSA/434-499
163 Metazoa_Q6B344_CHICK/253-318
164 Metazoa_Q66JF6_ENTR/36-100
165 fungi_Q5BC4_EMENI/197-262
166 Metazoa_H2ZUH7_LATCH/253-318
167 plant_AN4_FRAAN/86-151
168 Metazoa_H0ZZ00_TAEGU/95-160
169 plant_C6TNJ9_SOYBN/87-151
170 plant_A4S542_OSTLU/73-140
171 plant_B9T8F3_RICCO/1-34
172 plant_B6TT93_MAIZE/170-236
173 Stramenopiles_G4RU0_9STRA/1-37
174 plant_C54R1_SORBI/181-238
175 plant_AN4_FRAAN/244-309
176 Metazoa_B3RPM7_TRIAD/173-239
177 Metazoa_G4VI64_SCHMA/200-265
178 Metazoa_E1ZYA7_CAMFO/25-90
179 Stramenopiles_H3HB96_PHYRM/183-247
180 human_E5RK69_HUMAN/71-113
182 Metazoa_H3CF99_TETNG/91-156
183 fungi_C5FNC5_ARTOC/171-236
184 Metazoa_H2KP47_CLOSI/500-566
185 Metazoa_E3TCL0_9TELE/251-316
186 plant_G8E493_ORYSI/85-152
187 Metazoa_F7GDF3_MONDO/248-313
188 Metazoa_H2Y7G3_CIOSA/423-488
189 Metazoa_G1T8F2_RABIT/118-180
190 Metazoa_F1QKN3_DANRE/246-311
191 plant_AND4_ARATH/245-314
192 Amoebozoa_F0ZZ05_DICPU/67-135
193 Metazoa_E9GWB8_DAPPU/33-97
194 Metazoa_A7RW1_NEMVE/1-58
195 Metazoa_B51G1_SALSA/249-314
196 Metazoa_E1FLW0_LOALO/344-409
197 plant_A5B479_VITVI/170-235
198 plant_A5CB60_VITVI/289-332
199 Other_O_F2UI00_SALS5/434-500
200 Metazoa_E4KK2_OIKDI/81-144
201 plant_C0L7E4_ANNCH/246-311
202 Metazoa_G4VL68_SCHMA/20-85
203 Metazoa_H0ZQC9_TAEGU/20-85
204 Metazoa_G7Y5I1_CLOSI/626-692
205 Metazoa_G1MRM3_MELGA/443-483
206 Metazoa_B5RI22_SALSA/362-427
207 Metazoa_C1BKB4_OSMMO/192-258
208 Metazoa_C1BZS7_ESOLU/22-87
209 plant_C1E164_MICSR/592-654
210 Metazoa_F6ZVA4_ORNAN/1-32
211 Metazoa_AN13_CANFA/249-314
212 Metazoa_I1G0P6_AMPQE/238-303
213 plant_D7MT72_ARALL/15-80
214 Metazoa_B2L3Y5_ENBO/1-36
215 plant_G7LF83_MEDTR/87-152
216 Metazoa_G1PG64_MYOLU/117-182
217 plant_A9RBN7_PHYPA/14-79
543 Metazoa_O93447_ORYLA/279-344
544 bacteria_Fl_392964889.19
545 Metazoa_H2LTW7_ORYLA/266-331
546 Metazoa_C1C3N0_LITCT/45-110
548 Metazoa_E9H189_DAPPU/266-331
549 fungi_G1WYE9_ARTOA/427-494
550 plant_D7MT72_ARALL/87-152
551 Metazoa_G3USL9_MELGA/1-43
552 plant_F2DQP5_HORVD/87-152
553 plant_A9NMW6_PICSI/171-237
554 Metazoa_Q7QM48_ANOGA/19-84
555 fungi_B2B343_PODAN/433-500
556 Metazoa_F6U5G3_ORNAN/184-250
557 Metazoa_G4VI62_SCHMA/30-95
558 Metazoa_B2LU0_SCHBO/110-183
559 Metazoa_Q641H0_ENLA/432-497
560 Metazoa_H2SEN2_TAKRU/247-307
561 Metazoa_B5FE8_TAEGU/169-235
562 Metazoa_F6VPS5_MONDO/444-509
563 Metazoa_H0YUM4_TAEGU/86-151
564 Metazoa_C4WSW4_ACYPI/22-87
565 plant_I1IQN4_BRADI/81-149
566 Metazoa_H2VK4_CAEJA/19-81
567 Metazoa_H9GFN4_ANOCA/22-88
569 plant_Q56D10_TOBAC/247-312
570 Metazoa_H2Y7G3_CIOSA/347-413
571 Metazoa_B51G1_SALSA/90-155
572 fungi_G2Y61_BOTF4/170-235
573 Metazoa_F7B9R9_MONDO/87-152
574 fungi_E5ADB2_LEPMJ/200-265
575 fungi_Q5B3S2_EMENI/1315-1369
576 Other_O_F2UCQ3_SALS5/545-610
577 fungi_F9G7F4_FUSOF/343-407
578 Metazoa_O93444_ORYLA/20-85
579 Metazoa_A4QNM7_ENTR/251-316
580 Metazoa_F7B20_ENTR/285-350
581 plant_Q2TE7_SOLTU/245-310
582 plant_B9RGC8_RICCO/86-150
583 plant_Q6L4C5_ORYSJ/136-207
584 Metazoa_B2D0I4_ARTSF/330-396
585 fungi_A8N3U1_COPC7/356-431
586 Amoebozoa_F0YFK0_AURAN/76-141
587 Amoebozoa_F4Q2W8_DICFS/480-547
588 Metazoa_G1N2K9_MELGA/229-264
589 fungi_G0RCF8_HYPJQ/387-454
590 Metazoa_A0F047_PSEM/114-165
591 Metazoa_G3N8C9_GASAC/54-119
592 Metazoa_G6CV8_DANPL/25-90
593 Metazoa_H0P88_OTOGA/90-155
594 Metazoa_G3Q1P5_GASAC/46-111
595 Metazoa_H3APK8_LATCH/117-182
596 Metazoa_D3TLB6_GLOMM/91-156
597 Metazoa_F1NNR3_CHICK/252-317
598 Metazoa_G1KLU8_ANOCA/272-337
599 plant_A5AIA0_VITVI/17-82
600 bacteria_Mst_442322805.1
601 plant_Q1RUA5_MEDTR/173-238
602 Metazoa_C1C3N0_LITCT/117-182
603 fungi_B2VUR2_PYRTR/165-230
604 plant_B6SW41_MAIZE/26-60
605 plant_E5GCK5_CUCME/51-116
607 Metazoa_A8WG37_DANRE/170-236
609 Metazoa_H3IAM2_STRPU/18-83
610 Metazoa_Q6QAZ9_CHICK/272-337
611 plant_B9I5V5_POPTR/17-64
612 fungi_C7YG0_NECH7/373-440
613 Metazoa_G5ATS2_HETGA/148-214
614 plant_D3B4Z8_POLPA/387-454

218 fungi_C5GDZ2_AJEDR/316-380
219 Metazoa_C0H815_SALSA/17-82
220 Metazoa_F6WFY8_ORNAN/437-502
221 plant_I1IQN4_BRADI/167-234
222 Other_O_A9VD41_MONBE/1-64
224 Metazoa_H2M2M0_ORYLA/108-173
226 plant_I1HFK2_BRADI/15-80
227 fungi_G0SAF7_CHATD/405-453
228 Metazoa_E9J326_SOLIN/370-436
229 Metazoa_Q8WPH0_BOMMO/417-482
230 fungi_A2QCY7_ASPNC/380-447
231 Metazoa_ANB9_DROME/256-321
232 Metazoa_F6Y46_MONDO/599-664
233 Metazoa_G3Q341_GASAC/89-154
234 Metazoa_H0UWN6_CAVPO/45-110
235 Metazoa_B3P2Z0_DROER/97-162
236 Metazoa_D6WZQ0_TRICA/186-252
237 Metazoa_F7EBA7_ENTR/380-445
238 fungi_G0RVR1_HYPJQ/209-273
239 fungi_G2R268_THITE/416-483
240 plant_F2D6P4_HORVD/36-101
241 plant_F2E2N7_HORVD/143-209
242 fungi_B8M4F7_TALSN/637-704
243 plant_B9N394_POPTR/170-236
244 Metazoa_D3TME1_GLOMM/91-156
245 Metazoa_C3ZND3_BRAFL/71-137
246 Metazoa_F1P2N4_CHICK/93-158
247 Ecavates_GIA1_GIAIN/5-69
248 fungi_G2WWB5_VERDV/637-706
249 fungi_G3JNQ8_CORMM/214-279
250 plant_G7LEK9_MEDTR/51-118
251 plant_D8RJM2_SELML/28-66
252 fungi_E9DZQ0_METAQ/291-363
253 plant_B9SIA1_RICCO/85-151
254 plant_A9TFB3_PHYPA/14-79
255 Metazoa_O57570_DANRE/112-177
256 plant_B8AS2_ORYSI/94-145
257 Metazoa_ANA5_CHICK/19-84
258 plant_B9SM17_RICCO/86-151
259 Metazoa_H2TYU9_TAKRU/268-333
260 Other_O_E9C3C5_CAPO3/829-894
261 plant_G7IW11_MEDTR/86-151
262 plant_O24132_TOBAC/87-152
263 Other_O_E9CFN1_CAPO3/387-453
264 Metazoa_F1L0T9_ASCSU/20-85
265 Stramenopiles_D0NJ67_PHYIT/259-323
266 plant_A6N0R1_ORYSI/37-102
267 fungi_E7A392_SPORE/283-348
268 Metazoa_C1K7M5_ICTPU/336-402
270 Metazoa_G7PF12_MACFA/421-486
271 Metazoa_B0WMU9_CULQU/252-317
272 Metazoa_F1SS97_PIG/48-113
273 Metazoa_E9H188_DAPPU/266-331
274 Metazoa_H2M051_ORYLA/90-155
275 bacteria_Cc_380728269.21
276 Metazoa_B511_SALSA/18-83
277 Metazoa_A0SZ5_PERPL/1-29
278 fungi_A1C7G9_ASPCL/298-364
279 Metazoa_D3TME1_GLOMM/250-315
280 Metazoa_E1JJR8_DROME/91-156
281 plant_AND3_ARATH/15-80
282 Metazoa_C3ZND5_BRAFL/6-39
283 Metazoa_Q56J74_ONCTS/39-103
284 Metazoa_F6TS8_MONDO/91-156
285 fungi_Q6C9U3_YARLI/144-209
286 Metazoa_F6V1V9_ORNAN/257-322
287 Metazoa_H0LG9_OTOGA/267-332
288 plant_C6TD05_SOYBN/171-219

615 Metazoa_B3GQS3_9PLAT/285-350
616 Metazoa_A5LHA3_LITCT/39-104
617 fungi_F2T3D2_AJEDA/230-295
618 plant_D7M4J0_ARALL/14-79
619 plant_C5YL20_SORBI/87-152
620 Other_O_E9C3C5_CAPO3/901-966
621 Metazoa_C1C3N0_LITCT/276-341
622 fungi_Q0CY55_ASPTN/218-280
623 Metazoa_D1M7K2_ICTPU/91-156
624 Other_O_A9V2E0_MONBE/624-688
625 Metazoa_B2L46_ENBO/1-51
626 fungi_F2Q3M6_TRIEC/228-291
627 Metazoa_C1BIH9_OSMMO/17-82
628 fungi_D5G497_TUBMM/224-289
629 Metazoa_H9HRA6_ATTCE/256-321
630 plant_Q9ZRU7_CAPAN/87-152
631 fungi_D5G497_TUBMM/152-217
632 Metazoa_E9GSS2_DAPPU/265-330
633 Metazoa_D6WZQ5_TRICA/248-313
634 Amoebozoa_F4PN43_DICFS/385-452
635 fungi_E3RN47_PYRTT/650-687
636 Metazoa_F1L8I2_ASCSU/123-188
637 Stramenopiles_D0P3M8_PHYIT/154-220
638 Metazoa_E3TFU8_ICTPU/36-101
639 plant_B8BCJ7_ORYSI/246-312
640 bacteria_Sl_284035615.20
641 Metazoa_G3WWE4_SARHA/20-85
642 Metazoa_H2ZUH7_LATCH/94-159
643 Metazoa_B3S9E5_TRIAD/1-57
644 Metazoa_F6WVJ1_ORNAN/20-84
645 Metazoa_Q4VSV4_MONAL/268-333
646 Metazoa_E1GJQ8_LOALO/95-160
647 Stramenopiles_B5Y5D2_PHATC/27-92
649 plant_Q9FUG5_CERRI/170-235
650 Other_O_A9V4C6_MONBE/179-245
651 Metazoa_E9H187_DAPPU/26-91
652 Metazoa_E2RQ29_CANFA/201-268
653 Stramenopiles_G5A7E6_PHYSP/26-91
654 Metazoa_G3QPT3_GORGO/277-342
655 Metazoa_F1L0A1_ASCSU/347-413
656 Metazoa_Q4SM21_TETNG/267-332
658 fungi_E9DZQ0_METAQ/374-441
659 Metazoa_F1L0A1_ASCSU/192-257
660 fungi_B6GYL8_PENCW/398-465
661 plant_B8BF46_ORYSI/280-345
662 fungi_G0SAF7_CHATD/227-295
663 plant_C7EQ1_ORYSI/2-50
664 fungi_G3JNQ8_CORMM/373-441
665 fungi_Q2UFJ4_ASPOR/312-376
667 Metazoa_F6IA10_DANRE/515-581
668 Metazoa_G1KLU8_ANOCA/41-106
669 Metazoa_G3USL9_MELGA/50-90
670 Metazoa_G1KFA2_ANOCA/172-238
671 Metazoa_F1L2C7_ASCSU/175-241
672 Metazoa_F6WFY8_ORNAN/365-430
673 plant_A9NNL2_PICSI/170-234
674 plant_B9I5V5_POPTR/86-152
676 Metazoa_Q9GNG6_BOMMO/256-321
677 Metazoa_D3TM26_GLOMM/250-315
678 plant_I1HJW7_BRADI/104-153
679 plant_O22341_9ROSI/15-80
680 Metazoa_G3I5L3_CRIGR/201-267
681 Metazoa_G7Y5I1_CLOSI/542-608
682 Metazoa_H3BDR7_LATCH/108-174
683 Metazoa_A7RW3_NEMVE/88-153
684 Metazoa_H3BGL6_LATCH/437-502
685 plant_D7KVS1_ARALL/87-152
686 Metazoa_H2TGM1_TAKRU/173-239

289 fungi_Q2UFJ4_ASPOR/154-219
290 bacteria_Fb_363580181.14
291 plant_Q6S9D8_WHEAT/170-236
292 Stramenopiles_G4Z1N8_PHYSP/27-92
293 fungi_A8N3U4_COPC7/271-335
294 plant_F2DIL8_HORVD/207-272
295 Metazoa_D6WZQ1_TRICA/316-370
296 human_AA2L_HUMAN/37-102
297 Metazoa_H3IAM2_STRPU/175-241
298 Metazoa_F6ZL59_CALJA/433-495
299 Metazoa_G3NNR3_GASAC/90-155
300 fungi_Q4P8I3_USTMA/196-261
301 Metazoa_Q6P603_DANRE/267-332
302 plant_G7IW12_MEDTR/115-180
303 Metazoa_H2LS05_ORYLA/17-82
304 Metazoa_H3ETP9_PRIPA/172-238
305 Metazoa_D3PI40_9MAI/267-331
306 plants_D7FJE4_ECTSI/607-672
307 Metazoa_F8RBD9_SPIER/104-176
308 bacteria_Cc_380729140.22
309 Metazoa_C0H815_SALSA/89-154
310 Metazoa_H9GG64_ANOCA/318-384
311 Metazoa_A8SY2_CAEBR/170-235
312 Stramenopiles_D0NJ65_PHYIT/27-92
313 plant_F6KLJ6_ARAHY/14-79
314 fungi_H1VE74_COLHI/160-225
315 Metazoa_H2S105_TAKRU/118-183
316 Metazoa_F6QS6_MACMU/263-328
317 fungi_I1RBQ2_GIBZE/380-447
318 Metazoa_Q8ITJ0_HETGL/107-171
319 Metazoa_Q5F321_MANSE/256-321
320 Metazoa_F6YYM0_MONDO/201-264
321 Metazoa_E3TDF3_9TELE/246-311
322 Metazoa_H2KP47_CLOSI/344-409
323 Metazoa_H2PD2_CIOIN/60-125
324 Metazoa_B5CE7_SALSA/92-157
325 Metazoa_G1KFA2_ANOCA/17-82
326 Metazoa_ANA9_MOUSE/205-264
327 Metazoa_F1LDR9_ASCSU/99-164
328 Metazoa_H3IAM2_STRPU/90-157
329 Metazoa_Q9NGU7_TAESO/201-267
330 bacteria_Cc_380729140.2
331 bacteria_Cc_380728269.3
332 bacteria_Bb_375105645.4
333 Metazoa_G1NJ32_MELGA/181-247
334 bacteria_Ho_262194969.5
335 bacteria_Ho_262194969.6
336 Metazoa_E1FYF7_LOALO/22-87
337 plant_B9I5V5_POPTR/170-238
339 Metazoa_A1L514_BOVIN/90-132
340 Metazoa_F6ZAK1_HORSE/22-87
341 Metazoa_Q17A53_AEDAE/93-158
342 Metazoa_A5LHA6_LITCT/20-85
343 Metazoa_G1KJG0_ANOCA/86-151
344 Metazoa_C3ZND4_BRAFL/177-219
345 Metazoa_G0MLG0_CAEBE/281-346
346 Amoebozoa_ANA7_DICDI/165-230
347 Metazoa_H0ZCD8_TAEGU/257-322
348 Metazoa_Q9ZL9_TAESO/278-343
349 plant_D2D2Z9_GOSHI/244-309
350 Metazoa_D3TRS6_GLOMM/180-246
351 Metazoa_A2BFV8_DANRE/113-178
352 Metazoa_Q4VSV4_MONAL/192-258
353 Metazoa_Q804G8_DANRE/267-332
354 plant_A5BQ4_VITVI/96-155
355 plant_A5BQ4_VITVI/176-240
356 fungi_Q5BC4_EMENI/269-334
357 Metazoa_B2L3Y2_ENBO/193-259
687 plant_B7FJY0_MEDTR/14-79
688 Metazoa_F7H9W2_CALJA/119-179
689 plant_F2DV98_HORVD/256-321
690 Metazoa_A5LHA5_LITCT/174-240
691 Metazoa_B3RPM7_TRIAD/90-155
692 plant_D7T2Q7_VITVI/15-80
694 Metazoa_E9H189_DAPPU/188-256
695 Stramenopiles_F0WGW1_9STRA/185-251
697 plant_A5BTZ8_VITVI/238-303
698 Metazoa_G1N762_MELGA/62-118
699 plant_P93157_GOSHI/86-151
700 Metazoa_E2BIN4_HARSA/25-90
701 Metazoa_A87I3_CAEBR/266-331
702 fungi_C7YG0_NECH7/213-278
703 Metazoa_Q4ZGZ6_TAESO/243-308
704 fungi_F974_MYCGM/144-209
705 Metazoa_E0V9K1_PEDHC/207-272
706 Metazoa_A8E7L2_DANRE/1-38
707 plant_F2D6P4_HORVD/197-261
708 bacteria_Ms_409197576.31
709 plant_A9RZN4_PHYPA/199-264
710 Metazoa_Q6QAZ9_CHICK/196-262
711 fungi_Q0V6M8_PHANO/436-488
712 plants_D8LG01_ECTSI/11-77
713 plant_E5GCK3_CUCME/401-466
714 Ecavates_ANE1_GIAIN/21-78
715 Metazoa_G1SNT8_RABIT/24-89
716 plant_B4FZ06_MAIZE/64-129
717 Metazoa_A7RW3_NEMVE/172-238
718 fungi_Q6PKY9_ASPFM/313-379
719 Metazoa_C0H996_SALSA/250-315
720 Metazoa_G7PCS1_MACFA/125-191
721 Metazoa_G3ST95_LOAF/93-158
722 Other_O_A9VC1_MONBE/442-508
723 fungi_G9NI54_HYPAI/146-213
724 Metazoa_F6TU75_MONDO/15-80
725 Metazoa_H2PD2_CIOIN/142-208
726 Metazoa_G1NVK9_MYOLU/58-123
727 Metazoa_H9H3J1_MACMU/108-171
728 plant_B3TLL7_ELAGV/246-311
729 Metazoa_Q7ZM2_ENLA/270-335
730 Metazoa_F5HJB2_ANOGA/97-162
731 plant_Q4ABP7_BRARP/87-152
732 Metazoa_G3MPT6_9ACAR/336-401
733 fungi_G9N6Y4_HYPVG/309-374
734 plant_B9SM17_RICCO/14-79
735 Metazoa_G3PWL0_GASAC/182-248
736 Metazoa_F6IA10_DANRE/433-498
737 Metazoa_H2RYZ8_TAKRU/109-174
738 Metazoa_Q641H0_ENLA/273-338
739 Metazoa_E1ZYA7_CAMFO/256-319
740 Metazoa_B5BM7_SALSA/268-333
741 Metazoa_F1L0T9_ASCSU/92-157
742 Metazoa_C3ZQF7_BRAFL/237-303
743 Metazoa_D3Z0S1_MOUSE/75-133
744 Metazoa_E1FYF7_LOALO/177-243
745 plant_A5B79_VITVI/250-316
747 fungi_E4URJ3_ARTGP/312-378
748 plant_A9SPZ1_PHYPA/14-79
749 bacteria_Mm_124003075.8
750 Ecavates_Q8ITF7_GIAIN/154-220
751 Metazoa_H6BD08_OSTED/70-136
752 Metazoa_G3STG5_LOAF/117-182
753 plant_B9GWL9_POPTR/231-296
754 Metazoa_E9H191_DAPPU/101-166
756 Metazoa_B3GQS3_9PLAT/34-99
757 Other_O_F2TB8_SALS5/514-579
760 Metazoa_D3TME1_GLOMM/175-240

358 Metazoa_Q6DI3_ENTR/38-103
359 fungi_B6Q7Z7_PENMQ/376-443
360 plant_O24132_TOBAC/243-308
361 plant_Q42922_MEDSA/163-232
362 plant_C6TFT8_SOYBN/15-80
363 fungi_C0ND81_AJECG/253-317
364 Metazoa_B7QC21_IOSC/1-57
365 Metazoa_F1P6B7_CANFA/201-267
366 Metazoa_B4L73_DROMO/251-316
367 Metazoa_Q70KQ6_CIOIN/504-569
368 Metazoa_Q8MJB5_RABIT/214-280
369 Metazoa_A0F047_PSEM/38-104
370 Metazoa_E9H189_DAPPU/100-165
371 plant_Q69DC2_GOSHI/87-152
372 plant_G7LF84_MEDTR/14-79
373 fungi_A8N3U1_COPC7/203-257
374 plant_Q69DC2_GOSHI/170-235
375 plant_B9RJJ1_RICCO/245-310
376 Metazoa_F6YG66_CIOIN/323-388
377 plant_D7SPZ0_VITVI/13-77
378 Stramenopiles_G4Z1N4_PHYSP/128-176
379 Metazoa_Q6DKC4_ENLA/38-103
380 Other_O_A9VC1_MONBE/824-889
381 plant_I1MIU3_SOYBN/87-152
382 Metazoa_B4JK98_DROGR/91-156
384 Stramenopiles_H3HB96_PHYRM/98-163
385 Metazoa_A7RW2_NEMVE/74-131
386 plant_C5YL20_SORBI/15-80
387 Amoebozoa_F0ZZ7_DICPU/182-247
388 Amoebozoa_F1A6F3_DICPU/84-146
389 Metazoa_ANA5_CYNPY/93-158
390 Metazoa_G1MU27_MELGA/92-157
391 plant_D7U8J8_VITVI/163-230
392 Metazoa_G3NNS5_GASAC/178-244
393 Metazoa_F6YYM0_MONDO/117-182
394 Ecavates_C6LSD2_GIAIB/79-134
395 Stramenopiles_D0NJ65_PHYIT/260-324
396 Metazoa_A5LHA3_LITCT/194-260
398 plant_D3BHS8_POLPA/253-317
399 Metazoa_D6WZQ2_TRICA/319-385
400 plant_I1M0U7_SOYBN/86-151
761 Metazoa_E9H189_DAPPU/28-93
762 plant_B9SNI5_RICCO/84-149
763 plant_E5GCK3_CUCME/329-394
764 bacteria_Cc_380728269.10
765 Metazoa_ANA8_RAT/257-322
766 fungi_E9QB9_ASPFU/229-294
767 Metazoa_C1C3Z2_LITCT/177-243
768 Metazoa_G6D7C8_DANPL/331-397
769 Metazoa_A5LHA5_LITCT/19-84
770 Stramenopiles_F00L8_9STRA/185-251
771 Metazoa_F6YNZ9_MACMU/18-83
772 plant_B9RGD0_RICCO/174-239
773 Metazoa_H2PH43_PONAB/231-297
774 Metazoa_D3ZHD1_RAT/21-86
775 plant_I1P4D4_ORYGL/15-80
778 fungi_A2QCY7_ASPNC/304-370
779 plants_D8LG01_ECTSI/514-579
780 plant_D7M4J0_ARALL/244-309
781 Metazoa_AN10_MOUSE/93-158
782 Metazoa_G7MDI7_MACMU/201-264
783 fungi_G3JNQ8_CORMM/142-207
784 plant_G7LE90_MEDTR/15-80
785 fungi_E9D857_COCPS/373-440
786 plant_D7KL8_ARALL/246-311
787 Metazoa_A4QNM7_ENTR/175-241
788 Metazoa_G5AJY3_HETGA/269-334
789 fungi_G2WZN6_VERDV/196-264
790 bacteria_Fb_363580180.12
791 bacteria_Ka_163756662.13
792 Metazoa_G6D9T5_DANPL/93-158
793 Metazoa_E9GPJ9_DAPPU/164-229
794 bacteria_Aa_402495857.16
795 bacteria_Ch_110637878.17
796 bacteria_Fi_441497853.18
797 Metazoa_G3PZS3_GASAC/179-245
798 Stramenopiles_D0NJ67_PHYIT/97-163
799 bacteria_Ka_163756728.15
800 Metazoa_F6TF12_MONDO/400-465
801 bacteria_Bb_375105645.23
802 bacteria_Cc_380729140.24
803 bacteria_Ri_384102706.25
804 bacteria_Bb_375105645.26
805 bacteria_Ga_226228830.27
806 bacteria_Ga_226228830.28
808 Other_O_F2UBS2_SALS5/236-301
809 bacteria_Cc_380728269.29
810 Metazoa_F7IN55_CALJA/97-139
812 Metazoa_A7RW1_NEMVE/76-142
813 Other_O_F2UJT2_SALS5/505-570
814 Metazoa_F1MU06_BOVIN/201-264
815 bacteria_Ri_384102706.32
816 bacteria_Nm_258651831.33
817 Metazoa_H3JCA7_STRPU/33-87
