# Supplementary material for: Bioinformatics Analysis of Bacterial Annexins – Putative Ancestral Relatives of Eukaryotic Annexins
Source: PLoS One. 2014 Jan 16;9(1):e85428. doi: 10.1371/journal.pone.0085428 (PMC3894181; doi:10.1371/journal.pone.0085428)
Supplement: Figure S3 — HHpred results of sequence similarity analysis of wider regions of bacterial annexin proteins, showing weak sequence similarities to annexin tetrads. (PDF) [file pone.0085428.s003.pdf]

```
Hhpred query:
>gb|AFE05142.1|gi|380729140:960-1510 hypothetical protein COCOR_03294 [Corallococcus coralloides DSM 2259]
Hhpred top hit:
>1m9i_A Annexin VI; calcium-binding, membrane-binding, phosphorylation, mutant T356D, lipid binding protein;
  2.65A {Homo sapiens} SCOP: a.65.1.1 a.65.1.1 PDB: _1avc _A
  Probab=100.00  E-value=9.8e-80  P-value=1.9E-84  Score=691.32  Aligned_cols=381  Identities=22%  Similarity=0.302
Sum_probs=0.0

  Q ss_pred                CCCCCCCCHHHHHHHHHHHhcCCCCCHHHHHHHHHhcCCHHHHHHHHHHHHHHHHCcCHHHHHHHhcCchHHHHHHHHhhhhh
  Q gb|AFE05142.1|     4 QEPLFSNRARNISLKVWEGVDQWGTDEKKIFDALANLNPLQGHYIEVLYEKEHGQNLRERLKSELDDFFSTTTHDWDKAE   83 (551)
  Q Consensus          4 ~~~~F~~~~~~~DA~~L~kAikG~GtDE~~LieIL~~Rs~~Qr~~I~~aYk~~ygkdL~~~LkselsG~f~~lL~~~~R~~   83 (551)
                         +.|+||  |..||+.|++||+|+||||++||+||++||+.||++|+.+|+..||++|.++|+++++|+|+.++      +
  T Consensus         15 ~~~~fd-----Da~~L~kA~kG~GtDE~~iI~IL~~rs~~QR~~I~~~Y~~~~gkdL~~~LkselsG~fe~~l------~   86 (672)
  T 1m9i_A            15 DFPGFD--PNQDAEALYTAMKGFGSDKEAILDIITSRSNRQRQEVCQSYKSLYGKDLIADLKYELTGKFERLI------V   86 (672)

  Q ss_pred                HHhhcCCHHHHHHHHHHHhhhccccccCCCCCHHHHHHHHhcCCHHHHHHHHHHHHHHHHhCCcHHHHHhhhccCchHHHHHH
  Q gb|AFE05142.1|    84 ALLSGNAAKAAAVELNESMEGIFFGLGWGTDEAAILTLLRGKTPEQIEAIKKAYQQKYGKDLVVQMDDELRSGAFQTRDV  163 (551)
  Q Consensus         84 al~~~~~~~~~DA~~L~kA~kG~~~~~~~~~GtDe~~LieIL~~RS~~q~~~Ik~aY~~~YgkdLe~dIkseltSG~f~~~l~  163 (551)
                         +|+.+ |+++||..|++||+|.       |||+.+||||||+||||.|+++|+++|+++|+..||++|+++|+++ +||+|+++++
  T Consensus         87 ~l~~~~p~~~dA~~l~~A~kg~------gTde~~LiEIl~~Rsn~ei~~ik~aY~~~y~~sLe~di~~d-tsg~f~kll~  158 (672)
  T 1m9i_A            87 GLMRP-PAYCDAKEIKDAISGI------GTDEKCLIEILASRTNEQMHQLVAAYKDAYERDLEADIIGD-TSGHFQKMLV  158 (672)

  Q ss_pred                HHHHHH------HhcCcHHHHHHHHHHHHHhhcccccCCCCChHHhhHHHhhhcchhhhhhhhhccchhhhhhhhhhHHHHH
  Q gb|AFE05142.1|   164 DEMHAL------VEGRTEDARAIEMDRAMRGGWFFGLGTDRKAMEGVYDRIRKETEADAQQYGWDSKTFRAELLKRNQAI  237 (551)
  Q Consensus        164 ~~l~~l------~~~~~~~~~~~DA~~L~~A~~~~~~gkG~GTDe~~Li~Il~~~~~~~~~~~sRsnr~~~s~~hl~~ei~~~l~~I  237 (551)
                         .++.+-      +....++.||..|++|++++||    ||+..+|+||||        +|         |.+||          ++|
  T Consensus        159 ~ll~~~R~e~~~vd~~~a~~Da~~L~~Age~~~g----tde~~fi~IL~~~~~~~~~~~~R------S~~~L~~~~~~~~~~~~~v  213 (672)
  T 1m9i_A           159 VLLQGTREEDDVVSEDLVQQDVQDLYEAGELKWG----TDEAQFIYILG-------NR------SKQHL---------RLV  213 (672)

  Q ss_pred                HHHHHHHhCcCHHHHHhhcccccchhhhhhhhHHHHHhhcccccccccHHHHHHHHHHhccCChHHHHHHHHhCcHHH
  Q gb|AFE05142.1|   238 DASYEKQYGPDWTQRTAGESALSAAFHDEMKGEDLKLIEGLRDDKGLQVDAARIRIEHTSVFYADDKVIRDAMSAQGKRQ  317 (551)
  Q Consensus        238 ~~aY~~~yg~sLe~~I~~et~lsg~~~~~~~~~~~~~~~l~~~~r~ed~~~vD~~~l~~~~~~~~~~~gtde~~f~~Il~~rs~~~  317 (551)
                         .++|++.||++|+++|++++  ||+|++++
  T Consensus        214 ~~~Y~~~~g~~l~~~I~~e~--sG~~~~~L----------------------------------------------  241 (672)
  T 1m9i_A           214 FDEYLKTTGKPIEASIRGEL--SGDFEKLM----------------------------------------------  241 (672)

  Q ss_pred                HHHHHHhhhhhHHHHHHhHHHHHHHHHHHHHhcCHHHHHHHHHHhhhhhhhhhcccCcChhhHhheeeeehhhhcchhhhhhc
  Q gb|AFE05142.1|   318 IADARRDGYLDIQERLDIERTLDEAKVKPASEGPLTEEDRRKGRISELEFLEKWSPEKVQARIAKERKEVDVKAKAQAQA  397 (551)
  Q Consensus        318 l~~y~~~~~~~~~l~~~~d~~~~l~~~~v~~~~~~~~~~fA~~l~~~~~~~~~~amkg~gT~~~~liri~vsR~e~d~~~~~~~~~~~~~  397 (551)
                                                 .++|+|+.|||.|||++||      +||+|+||+|++||||+|+|+|+
  T Consensus        242 -----------------------l~iv~~~~~~~~~~fA~~L~~~~~~~~amkg~GT~d~~LiRiivsR~e~~~~~~~~~~~~~~  283 (672)
  T 1m9i_A           242 -----------------------LAVVKCIRSTPEYFAERLF-----KAMKGLGTRDNTLIRIMVSRSEL----------  283 (672)
```

```
Q ss_pred               ccHHHHHHHHHHHHHHhCCCcccCCcHHHHHHhhCCHHHHHHHHHHHCCCC-----------------------------
Q gb|AFE05142.1|  398   DAPANMKALAAEYDSAYVEGTHLQATFEQAVLMDTSGADREAVAALLAKGY-----------------------------  448 (551)
Q Consensus       398   ~~~~dl~~Ik~~y~~~yg~~~~~~~~sL~~~I~~etSG~y~~~Ll~Ll~~~~-----------------------------  448 (551)
                            ||..||++|+++||       +||.++|+++|||||+++||+||+...
T Consensus       284   ----Dl~~Ik~~y~~~yg------~sL~~~I~~~tsGdY~~~LlaL~g~~d~~~g~~~pe~a~~a~~~~~~~~~~~~~~~~~  353 (672)
T 1m9i_A          284   ----DMLDIREIFRTKYE------KSLYSMIKNDTSGEYKKTLLKLSGGDDDAAGQFFPEAAQVAYQMWELSAVARVELK  353 (672)

Q ss_pred               -----------CcHHHHHHHHHhcCCCCHHHHHHHHhcCCHHHHHHHHHHHHhcccccccccchHH-HHHHHHHHHhcc
Q gb|AFE05142.1|  449   -----------LTDAEIVQYAVQGVGTDEDALKEVFAGKTKAEIQAMAEEWAKDHPADPGDTRTPFE-RFRARIDEELGG  516 (551)
Q Consensus       449   -----------~~~a~~l~~a~~g~gtde~~l~~i~~~~~~~~~~~~~~~~~~~~~~~~~~~~~~~~~~~~~~~~~~~~~~~~~~~~~g  516 (551)
                                   ..||+.|++||+|+||||.+||+||++||++|+|+|+.+|++           .|+ ||.++||||+||
T Consensus       354   ~tv~p~~~fd~~~Dae~L~kA~kG~GtDE~~iI~IL~~rs~~QR~~I~~~Y~~-----------~~gkdL~~~LkselsG  422 (672)
T 1m9i_A          354   GDVRPANDFNPDADAKALRKAMKGLGTDEDTIIDIITHRSNVQRQQIRQTFKS-----------HFGRDLMTDLKSEISG  422 (672)

Q ss_pred               chHHHHHHHHHhcCCCCChHH
Q gb|AFE05142.1|  517   REEFDILDMVDYGEPVTPRE  536 (551)
Q Consensus       517   ~~~~~~~~~~~~~~~~~~~~~~~~~~~~~~~~~~~~~~~~  536 (551)
                        +|+..|+.|+..--..++.+
T Consensus       423   ~fe~~l~~l~~~p~~~dA~~  442 (672)
T 1m9i_A          423   DLARLILGLMMPPAHYDAKQ  442 (672)
```

Hhpred query:
>gi|262194969:210-560 Annexin repeat-containing protein [Haliangium ochraceum DSM 14365]
Hhpred top hit:
>1yii_A Annexin A5, annexin V, lipocortin V, endonexin II; membrane binding, matrix vessicle, protein and metal binding protein; 1.42A {Gallus gallus} SCOP: a.65.1.1 PDB: 1yj0 _A 1ala _A 1hvd _A 1anx _A 1anw _A 1avh _A 1avr _A 1hak _B* 1hvf _A 1hve _A 1hvg _A 1a8a _A* 1a8b _A* 2ie7 _A 1g5n _A 2ie6 _A 1bcz _A 1n41 _A 1bcw _A 2ran _A ...
Probab=100.00 E-value=9.5e-73 P-value= 1.8E-77 Score=548.02 Aligned_cols=287 Identities=20% Similarity=0.278 Sum_probs=0.0

```
Q ss_pred               CCCCCCHHHHHHHHHHHhcCCCCCHHHHHHHHHHhCCCHHHHHHHHHHHHHHHCcCHHHHHHHHcCccHHHHHHHHHhhc
Q gi|262194969:2    1 TVRVVNVQALADRIHEAIDGLGTDEEAVYSALSALQHDADNIKGLRDAYQSKFGCSLDDDIRGDFSGDELSHALSLLAAK   80 (351)
Q Consensus         1 ~~~~fd~~~DA~~L~~A~kG~GtdE~~LieIL~~~~rs~~q~~~I~~~Y~~~yg~~L~~dI~~e~sG~~~~lll~ll~~~   80 (351)
                        +.++|||+.||+.|++||||+||||++||+||  |+|||+||++|+++|+..||++|+++|++++||+|++++++|+.
T Consensus        10 ~~~~f~~~~Da~~L~~A~kG~GtdE~~li~IL~~~~Rsn~q~~~I~~~Y~~~~g~dL~~~lkselsG~f~~~l~~l~~~~   85 (320)
T 1yii_A          10 AFSPFDARADAEALRKAMKGMGTDEETILKIL--TSRNNAQRQEIASAFKTLFGRDLVDDLKSELTGKFETLMVSLMR--   85 (320)

Q ss_pred               cCcccccccccccccccccHhhhccHHHHHHHHHHhcCCCCCHHHHHHHHHhCCHHHHHHHHHHHHHHHHhCCCHHHHh
Q gi|262194969:2   81 SAPAAGGAGAGDAAAGGGKAAAGLDYVALAKDIHEAVAGAGTDEEAVFAALSRLGGDRVKAAQLSGAYVGTYQTSLRSAL  160 (351)
Q Consensus        81 R~e~~~p~~~~~~~~~~~~~~vd~~~~e~~~DA~~L~~A~~g~gtde~~lieIL~~rs~~~l~~~~~I~~~Y~~~yg~sL~~~I  160 (351)
                             +|                  ++  +||+.|++|++|+||||.+||+|||+||+.|+++  |+++|++.||++|+++|
T Consensus        86 ----~~------------------------~DA~~L~~A~kg~GTde~~lieIL~~Rs~~ql~~~~I~~aY~~~y~~~Le~~I  140 (320)
T 1yii_A          86 -----PA----------------RI--FDAHALKHAIKGAGTNEKVLTEILASRTPAEVQN--IKQVYMQEYEANLEDKI  140 (320)

Q ss_pred               hhhcChhHHHHHHHHHHcccCccccccChHHHHHHHHHHHHHHhCcCCCCCHHHHHHHHH------HHHHHHHHHHhCCC
Q gi|262194969:2  161 SDDLDEDELNYALELLGDRQNGEMVNVANDEEAKKASAIIRRIYENYGIDVNSQAGVDA------IRRDYDEVPQSVRDS  234 (351)
Q Consensus       161 ~~e~sG~~~~~Ll~ll~~~r~~~~~~~vd~~~a~~dA~~L~~A~~g~~gtde~~lirIl------L~~I~~~Y~~~yg~s  234 (351)
                     ++++||+|+++|++|+++.|++ ..+||+.+|++||+.||+.|+.+.+||+..|++||+      |++|+++|++.||++
T Consensus       141 ~~e~SG~~~~~ll~ll~~~r~~~~~~~vd~~~a~~dA~~L~~A~~~~~~Gtde~~li~Il~~Rs~~~L~~i~~~Y~~~~~g~s  219 (320)
T 1yii_A         141 TGETSGHFQRLLVVLLQANRDP-DGRVDEALVEKDAQVLFRAGELKWGTDEETFITILGTRSVSHLRRVFDKYMTISGFQ  219 (320)

Q ss_pred               HHHHH-----HHHH-HHHHHHHHHhcChHHHHHHHHHHHHHhccCCChhHeeeEEEecccccccchhhHHHHHHHHHHHhC
Q gi|262194969:2  235 LKTTA-----WEYK-ELVALEKALARFAPILGSARGNSTRSGEGQEIRTVSKVDQAIDENSDAGVLDTTTLGEYFESSEN  308 (351)
Q Consensus       235 L~~ai-----ge~~~Llalv~~~~~~~~~~~~~~A~~l~~a~~g~gt~~~~l~ri~v~~~~~r~~~~e~dl~~Ik~~y~~~yg  308 (351)
                   |+++|      |+|+ .|+++|+|+ +|||.|||++||+|+|+|+++||||+|+     ||    |+||..||++|+++||
T Consensus       220 L~~~I~~~e~sGd~~~~Llalv~~~~~~~~~~~A~~L~~amkg~gt~~~~Liri~vs---Rs---e~dl~~Ik~~y~~~yg  292 (320)
T 1yii_A         220 IEETIDRETSGDLEKLLLAVVKCI-RSVPAYFAETLYYSMKGAGTDDDTLIRVMVS---RS---EIDLLDIRHEFRKNFA  292 (320)
```

```
Q ss_pred                 CchHHHHHHHHH-HhHHH
Q gi|262194969:2     309 FSMFTAGESST-IDFAD  324 (351)
Q Consensus          309 ~~L~~~i~~~t-~~~~~~  324 (351)
                         +||.++|+++| .||+.
T Consensus          293 ~sL~~~I~~~tsGdy~~  309 (320)
T 1yii_A             293 KSLYQMIQKDTSGDYRK  309 (320)
```

```
Hhpred query:
>gi|497236035:870-1360 Colicin D [Burkholderiales bacterium JOSHI_001]
Hhpred top hit:
>1m9i_A Annexin VI; calcium-binding, membrane-binding, phosphorylation, mutant T356D, lipid binding protein;
  2.65A {Homo sapiens} SCOP: a.65.1.1 a.65.1.1 PDB: _1avc _A
  Probab=100.00  E-value=4.2e-91  P-value= 8.1E-96  Score=771.27  Aligned_cols=425  Identities=22%  Similarity=0.290
Sum_probs=0.0


  Q ss_pred               CCCCCHHHHHHHHHHHhCCCCccccCCCCHHHHHHHHHcCCHHHHHHHHHHHHHHhCCCHHHHHHHHcCCCHHHHHHHH
  Q gi|497236035:8    3 IVGRDVNGLASNLYAAFHGNWGWLSEAGTDEDAVYAALDGLSRIQSLALRNVYRRRYGVELESELRSELSGGELDRAEAL   82 (491)
  Q Consensus         3 ~~~~d~~~DAe~L~kA~kG~~~~~~~~~GtDE~~LIeIL~~RS~~Qr~~Ir~aY~~~yg~dL~~~LkselSG~f~~lll~l   82 (491)
                        .|+|||..||+.|++||+|        +||||++||+||++||+.||++|+.+|+..||+||.++|++|+||+||++++++|
  T Consensus        16 ~~~fd~~~Da~~L~kA~kG-------~GtDE~~iI~IL~~rs~~QR~~I~~~Y~~~~gkdL~~~LkselsG~fe~~l~~l   88 (672)
  T 1m9i_A           16 FPGFDPNQDAEALYTAMKG-------FGSDKEAILDIITSRSNRQRQEVCQSYKSLYGKDLIADLKYELTGKFERLIVGL   88 (672)

  Q ss_pred               hcccHHHHHHHHHHHhhhcccccccCCCCHHHHHHHHHhCCHHHHHHHHHHHHHHhCcCHHHHHHHhcch-hhHhHh--h
  Q gi|497236035:8   83 LSGNRAEAAAAALYTAMHETFLGTGAGTDEATIHSTLRGLNAEERAEVTRIYRERYGIDLAGDVRGELDD-WATIGT--W  159 (491)
  Q Consensus        83 l~~pR~e~DA~~L~~AlkG~~~~~~~~~gTDe~~LieIL~tRS~~eL~~Ik~aY~~~yg~sLeedI~se~sG~rklLl--l  159 (491)
                        +++|  +++||..|++||+|       .|||+.+||||||+|||+||.+|+++|+..|++||++||.++|||  |+++|+  +
  T Consensus        89 ~~~p~~~~dA~~l~~A~kg------gTde~~LiEIl~~Rsn~ei~~ik~aY~~~y~~sLe~di~~dtsg~f~kll~~ll  161 (672)
  T 1m9i_A           89 MRPP-AYCDAKEIKDAISG------IGTDEKCLIEILASRTNEQMHQLVAAYKDAYERDLEADIIGDTSGHFQKMLVVLL  161 (672)

  Q ss_pred               hcccc-CCCCCHHHHHHHHHHHHHhhcccCCCCccHHHHHHHHHhccccccccccCCCHHHHHHHhhhhHHHHHHHHH
  Q gi|497236035:8  160 QADRA-DAELAGNLQAADAIELDEELRGSWFHSSTTTTVAAVYDRVRSEVQAQATREHRSSAWMEAEITRRTWGINTEYD  238 (491)
  Q Consensus       160 ~~~R~~~~~Vd~~~a~~DA~~L~~A~~~k~gt~~De~~~irIl~~i~~~l~~rv~R~~RS~~~L~a~~~~~~~~~~I~~~Y~  238 (491)
                        ++.|+ +.+||++.|+.||+.||+||+++++||  |++.|++||++                ||++||++          |+++
  T Consensus       162 ~~~R~e~~~vd~~~a~~Da~~L~~Age~~~gt~~de~~fi~IL~~~~~~~~~~~~~~~~~~RS~~~L~~~~~~~~~~~~v~~~~~~~  216 (672)
  T 1m9i_A          162 QGTREEDDVVSEDLVQQDVQDLYEAGELKWGT--DEAQFIYILGN-------------RSKQHLRL--------VFDE--  216 (672)

  Q ss_pred               HHhhhhhhh-HHHHHHHhcCCCcccccccchhHHHHHHHHHHHhcCCHHHHHHHHHHHHHhccCCCHHHHHHHHHHhhh
  Q gi|497236035:8  239 RRYAATSGT-LNQAIDAGTRAERNFWGNAPGNQEASADYLHALARNDMATADAARIRIERTSLVYADDDTITTTVGSQYN  317 (491)
  Q Consensus       239 ~~Y~~~~~G~~L~~aI~~e~sG~~~~~~~~~~~~~~~d~~~aLl~iv~~~~~p~~~fA~~L~~am~G~GT~d~~Li~Rvv~~~~~e  317 (491)
                          |++.+|+ |+++|+++|||           +++++|+++|+|+.|||.|||++||.||+|+||+|.+||  |||++|+|
  T Consensus       217 --Y~~~~g~~l~~~I~~e~sG----------------~~Ll~iv~~~~~~~~~~~~fA~~L~~amkg~GT~d~~Li-RiivsR~e  282 (672)
  T 1m9i_A          217 --YLKTTGKPIEASIRGELSG-----------DFEKLMLAVVKCIRSTPEYFAERLFKAMKGLGTRDNTLI-RIMVSRSE  282 (672)
```

```
Q ss_pred             hhHHHHHHHHHH----------------HHHHHHHhhccchhhhhhccCcch-----hhhhH----HHHhhhhhhhhhhh
Q gi|497236035:8  318 RALLAERLDYAP----------------ERRRALLAALDARERAEGRVWTPA-----ERWAA----QQEIERTIEHELIT  372 (491)
Q Consensus       318 ~~~~~~r~~~~~~~~~~~~~~~~~~~~~~~~r~~~~~~~~~~ra~~~~~~~~~~~~~~~~~~~~~~~~~~~~~~~~~~~~  372 (491)
                      .||..||.+|.+                .|+++||.+|++++-+.|+|||+.     +||++    ++...+||.|...-
T Consensus       283 ~Dl~~Ik~~y~~~yg~sL~~~I~~~tsGdY~~~LlaL~g~~d~~~g~~~pe~a~~a~~~~~~~~~~~~~~~~~~~~tv~p~~~f  362 (672)
T 1m9i_A          283 LDMLDIREIFRTKYEKSLYSMIKNDTSGEYKKTLLKLSGGDDDAAGQFFPEAAQVAYQMWELSAVARVELKGDVRPANDF  362 (672)


Q ss_pred             hhcCcH-------------------------HHHHHHHHHHHHHHhCCcHHHHHHhhcCCcHHHHHHHHHhccccCCHHH
Q gi|497236035:8  373 RARGTA-------------------------DANLARMESEYESRYGESARTAILDSTSGVSHDKASTMLDQGGYLDPYQ  427 (491)
Q Consensus       373 ~a~~d~-------------------------~~~~~~~i~~~y~~~y~~~l~~~v~~~~sG~y~~~l~~l~~~~~~~~~~~~~~~~  427 (491)
                      -+..|.                         ++||+.|+.+|+++||++|.+++++++||++++.++.|+..-...++ .
T Consensus       363 d~~~Dae~L~kA~kG~GtDE~~iI~IL~~rs~~QR~~I~~~Y~~~~gkdL~~~LkselsG~fe~~l~~l~~~p~~~dA-~  441 (672)
T 1m9i_A          363 NPDADAKALRKAMKGLGTDEDTIIDIITHRSNVQRQQIRQTFKSHFGRDLMTDLKSEISGDLARLILGLMMPPAHYDA-K  441 (672)

Q ss_pred             HHHHHhccCCCCHHHHHHHHhccCHHHHHHHHHHHHHHhcCCCHHHHhhccCCCc
Q gi|497236035:8  428 TFDYAVRGAGTDEAAATRAFAGLTAQEIARLDLRWQREHGGQTLRQAAASELSGR  482 (491)
Q Consensus       428 ~~~~~a~~g~gtde~~~~~~~~~~~~~~~ei~~~~~~~~~~~~~~~~~~~~~~~~~~~~~~  482 (491)
                      .+++||+|.||||.+||||||||||+||..|+.+|+..| |+|||+.|.|||||+
T Consensus       442 ~l~~A~kg~gTde~~LiEIL~~Rsn~ei~~Ik~aY~~~y-~~sLe~di~~dtsg~  495 (672)
T 1m9i_A          442 QLKKAMEGAGTDEKALIEILATRTNAEIRAINEAYKEDY-HKSLEDALSSDTSGH  495 (672)
```

Hhpred query:
>gi|226228830:1130-1500 hypothetical membrane protein [Gemmatimonas aurantiaca T-27]
Hhpred top hit:
>d2zhja_ a.65.1.1 (A:) automated matches {Norway rat (Rattus norvegicus) [TaxId: 10116]}

```
 Probab=96.56  E-value=0.0034  Score=55.11  Aligned_cols=187  Identities=20%  Similarity=0.174  Sum_probs=0.0

 Q ss_pred               cHHHHHHHHhhccccccceeeccCCCccHHHHHHHHHhcCcccccceEEEEEecCCCCHHHH-HHhcCCCcHHHHHHHHH
 Q gi|226228830:1  122 DMDALRAVFKSDYKLDLDEVVLSDTSGYDNQKAATLLKQGGHLTLGQTMFFAVRGNGTDEEAL-NALKGHTSEEIDEARA  200 (371)
 Q Consensus       122 dmdalravfksdykldldevvlsdtsgydnqkaatllkqgghltlgqtmffavrgngtdeeal-nalkghtseeideara  200 (371)
                      .+..++..|+..|.-||.+-+-+++|| +-+++...|-.+-.--..+.++-|++|.||||+++ ..|-+++++++.+.+.
 T Consensus        47 q~~~i~~~Y~~~~g~~L~~~i~~~~sG-~~~~~ll~~l~~~~~~~~~da~~L~~A~kg~gtde~~li~Il~~rs~~~~~~~~i~~  125 (315)
 T d2zhja_         47 QRQEIRTAYKSTIGRDLLEDLKSELSS-NFEQVILGMMTPTVLYDVQELRRAMKGAGTDEGCLIEILASRNPEEIRRINQ  125 (315)
 T ss_dssp            HHHHHHHHHHHHHCSCHHHHHHHHHCCH-HHHHHHHHHHSCHHHHHHHHHHHHHSSSCCCHHHHHHHHHHSCHHHHHHHHH
 T ss_pred            HHHHHHHHHHHHhCCCHHHHHHHHHcCc-cHHHHHHHHHhCChHHHHHHHHHHHhcCCCCCHHHHHHHHhcCCHHHHHHHHH


 Q ss_pred               HHHHHHHHhHHHHhhhhhhhhcCCCCccccccchhhcccccCccccHHHHhcCCCc--------hHHHHHHHHHHHhhhh
 Q gi|226228830:1  201 EFRQLALADRWTARGILNRAWSDRPHNDLDMDAEIKDDISGRTGFDVGQMLQGEPK--------TIREKRARLLEAITWE  272 (371)
 Q Consensus       201 efrqlaladrwtargilnrawsdrphndldmdaeikddisgrtgfdvgqmlqgepk--------tirekrarlleaitwe  272 (371)
                      .|++.                          ..-|+...|+.+.||-..-=+--+++|...        ..+.--..|.+|+  +
 T Consensus       126 ~Y~~--------------------~~~~L~~~i~~e~sg~~~~ll~~ll~~~r~~~~~~vd~~~~~~~~da~~L~~A~---~  182 (315)
 T d2zhja_        126 TYQQQ---------------------YGRSLEEDICSDTSFMFQRVLVSLTAGGRDEGNYLDDALVKQDAQDLYEAG--E  182 (315)
 T ss_dssp            HHHHH---------------------HSSCHHHHHHHHCCHHHHHHHHHHHHHCCCCCSCCCCHHHHHHHHHHHHHHT--T
 T ss_pred            HHHHH---------------------hCcCHHHHHHHhhcCccHHHHHHHHHhhccCCCCCCCcchHHHHHHHHHHHc--c


 Q ss_pred               hccCchhhhhhhhhhhhhhhhHHHHHHHHHHHhcCccccCHHHHhhhhhhhhhhhhhHHHHhHH
 Q gi|226228830:1  273 KEAGPLGDWLAGHQQSVMDADLERLDRTIAMLENPELSAEQRKRYIGYFEQDVTTVHAGI  332 (371)
 Q Consensus       273 keagplgdwlaghqqsvmdadlerldrtiamlenpelsaeqrkryigyfeqdvttvhagi  332 (371)
                      +..|+=.+-+.----+--..++.++.......-+-.|...-++..-|.|+.=+.+++.+.
 T Consensus       183 ~~~gtd~~~li~il~~rs~~~l~~I~~~Y~~~~~g~sL~~~I~~~~~sG~~~~~~Ll~ll~~~  242 (315)
 T d2zhja_        183 KRWGTDEVKFLSILCSRNRNHLLHVFDEYKRISQKDIEQSIKSETSGSFEDALLAIVKCM  242 (315)
 T ss_dssp            TSSSCCHHHHHHHHHHSCHHHHHHHHHHHHHHHHSSCHHHHHHHHCCHHHHHHHHHHHHHH
 T ss_pred            ccCCCCHHHHHHHHHhCCHHHHHHHHHHHHHHHhCCCHHHHHHHhcCchHHHHHHHHHHHhc
```
